# More Motivated to Help Male Leaders? Explaining Fatherhood Bonuses via Follower Helping

**DOI:** 10.1007/s10551-026-06248-1

**Published:** 2026-02-11

**Authors:** Jamie L. Gloor, Susanne Braun, Jenny M. Hoobler, Huong Pham, Claudia Peus

**Affiliations:** 1 University of St. Gallen School of Management, Dufourstrasse 40a, 9000 St. Gallen, Switzerland; 2 Durham University Business School, Durham University, Durham, UK; 3 NOVA School of Business and Economics, Universidade NOVA de Lisboa, Campus de Carcavelos, Carcavelos, Portugal; 4 THI Business School, Technische Hochschule Ingolstadt, Ingolstadt, Germany; 5 TUM School of Management, Technical University of Munich, Munich, Germany

**Keywords:** Work-family conflict, Leader gender, Followership, Motherhood bias, Fatherhood bonus

## Abstract

Family amplifies gender biases in organizations, presenting an ethical dilemma for modern organizations. Specifically, women tend to face additional barriers to leadership when they become mothers, while men often experience the opposite. But what about leaders’ family explains these fatherhood bonuses? Focusing on work-family conflict (WFC), we theorize that this ostensibly negative state paradoxically motivates followers because it signals an opportunity to expend extra effort to help their leaders. We further theorize this helping process is stronger for male (vs. female) leaders because WFC is a more noticeable violation of gendered stereotypes, which followers respond to with motivation to help their (male) leaders to resolve cognitive dissonance. A multi-source field study of leader–follower dyads (*N*_total_ = 290) supported our theorizing, which we causally replicated and extended with a pair of pre-registered behavioral and recall experiments (*N*_total_ = 920). These results inform theory and practice of gender inequalities while also revealing new mechanisms that explain behavioral fatherhood bonuses.

**Supplementary Information:**

The online version contains supplementary material available at https://doi.org/10.1007/s10551-026-06248-1.

Gender bias is a persistent and pervasive ethical concern for modern organizations. When it comes to women leaders, progress toward workplace equality has not only stalled (Caleo & Heilman, 2019), but has reversed (Elsesser, 2022), with explanations for this social problem including gender biases against women (Bernstein et al., 2020). Assessing whether gender bias plays a role in the careers of men and women leaders has direct ethical implications for organizations and contributes to United Nations Sustainable Development Goal (SDG) #5, promoting gender equality. Organizations are failing to follow through on creating fair, inclusive work environments (Bernstein et al., 2020; McKinsey & Company, 2021a, 2021b, 2021c). In response to this ethical conundrum, the management literature has called for more targeted research on gender bias to offer specific explanations why and how gender equality remains out of reach (Heilman & Caleo, 2018). Here, we develop and test the idea that followers’ knowledge of their leaders’ family responsibilities may be biased in ways that privilege and enhance the careers of male—but not female—leaders.

Family amplifies the potential ethical implications of gender biases in organizations: Women face additional barriers to leadership when they become mothers (see Arena et al., 2023; Grandey et al., 2020) while men often experience the opposite (e.g., fatherhood bonuses in pay and promotions; Glauber, 2008; Hodges & Budig, 2010; Kmec, 2011). Fathers in higher-status, masculine jobs (i.e., managerial workers) earn even bigger fatherhood bonuses (Hodges & Budig, 2010) without working more (Glauber, 2008). Thus, male leaders in white collar jobs reap the largest parenthood bonuses, contributing to gender inequalities and posing an important ethical matter for organizations to address.

But existing research relies on a downward direction of influence through which decision-makers notice and reward parenthood for men leaders. Critically, the micro-level literature examining the upward direction of influence—how followers view leaders who are parents—provides little evidence if others’ perceptions and reactions towards men and women leaders differ according to leaders’ parental status. For example, research by ten Brummelhuis et al. (2014) explored how leaders’ family status affects their followers, showing negative effects of family-work conflict and positive effects of family-work enrichment; yet, no gender differences were reported despite the mounting research relying on a downward direction of influence showing evidence of fatherhood bonuses, especially for those in higher-status, leadership roles (e.g., Glauber, 2008; Hodges & Budig, 2010; Kmec, 2011). At the same time, when men engage in fathering activities other than breadwinning, it can generate flexibility stigma (i.e., devaluation of childcare or family accommodations; Coltrane et al., 2013; Gloor et al., 2018) and other negative consequences among targets (e.g., increased stress and depression) and observers (e.g., penalization as lacking in masculine traits, aversive sexism) within the same hierarchical level in organizations (Gatrell et al., 2022; Ladge et al., 2015; Williams et al., 2013).

To make sense of these mixed findings, we focus on leaders, integrating motivational as well as moral theories to explain the moral implications of gender biases in organizations. We implicate motivation because family shapes leaders’ motivation of their followers (ten Brummelhuis et al., 2014). We also highlight the moral implications as well, namely, that observers’ perceptions of leaders’ work-family conflict involve judgments about the leader’s moral character, strongly influenced by leader gender. Work-family research has traditionally drawn from resource and role theories (e.g., Li e al., 2017b). Yet, to understand why work-family-related perceptions of others are sometimes associated with positive—and other times negative—career and well-being outcomes, employing more precise psychological theorizing to understand how work-family observations link to cognition and behavior is necessary (Carlson et al., 2023). As Li et al. (2017b) contend, a turn toward the leader–follower relationship in work-family scholarship is needed; they call for work that broadens the range of work-family research to acknowledge that followers observe and make attributions of leaders’ behavior that influence follower responses (Li et al., 2017b).

Integrating and building on motivational and moral theorizing, we propose that prosocial motivation—the desire to expend discretionary effort based on a concern for helping or contributing to another person’s aims and needs satisfaction (Grant, 2007)^1^—directs employees’ attention towards others’ perspectives, including what might be useful for them (Grant & Berry, 2011). We predict this other-focused psychological process is more strongly oriented towards leaders than other people who may be perceived to have WFC (e.g., coworkers or non-leaders) because leader–follower relationships are more salient in organizations (Martin et al., 2016, 2019) due to leaders’ higher status and powerful influence over followers’ work and nonwork lives (Braun & Peus, 2018; Hammer et al., 2011; Stollberger et al., 2019). We argue that organizations can be faced with an ethical conundrum when the pursuit of gender equality stalls because followers act with the best intentions, that is, to prosocially support their leaders who experience work-family conflict.

But what is it about leaders’ family that motivates their followers? Focusing on work-family conflict^2^—“*the most frequently studied construct in the work-family field*” (Kelly et al., 2010, p. 310)—we theorize that the ostensibly negative state of work-family conflict paradoxically motivates followers to expend extra effort to ‘help out’ (Kuvaas et al., 2012; van Dijke et al., 2018) the leader. Indeed, negative events evoke stronger cognitive responses which mobilize behavioral reactions to minimize or even erase the impact of the event on the target (Taylor, 1991). This process is particularly impactful when the leader is male, due to gender stereotypes that more closely associate women with communality and family, and men with agency and work (Eagly & Wood, 2012; Heilman, 2012)—especially leaders (Hentschel et al., 2018). In contrast, working women are stereotypically viewed as being in constant conflict between work and family domains (Grandey et al., 2020; Hoobler et al., 2009; Padavic et al., 2020). As a result, we theorize that followers react with a stronger response and desire to help high WFC male (vs. female) leaders to reduce their cognitive dissonance (between “family” and “man”)—an inherently motivating state (Festinger, 1962) because helping is a dissonance-reducing behavior (Harmon-Jones & Harmon-Jones, 2019). This motivation to reduce discomfort may be channeled through a primary means through which followers exert their autonomy at work: extra effort toward their work and/or their leader. Although this help may not alleviate leaders’ WFC, it stands to benefit those leaders and the organizations that employ them (Aw et al., 2021). However, this process is inherently problematic from an ethical perspective because, by taking prosocially motivated action, followers may aggravate issues of workplace inequality for women leaders. Because work-family studies have focused on self-reported WFC (e.g., Byron, 2005; French et al., 2018; Hoobler et al., 2010; King, 2008; Shockley et al., 2017; Trzebiatowski & Triana, 2020) or downward influence (i.e., supervisor ratings of follower WFC; Bagger et al., 2008; Hoobler et al., 2009, 2010; King, 2008; Steiner et al., 2023), this idea is ripe for theorizing and testing.

This research makes four core contributions to the work-family, ethics, and leadership literatures. First, we integrate motivational and moral theories to identify a novel phenomenon: an at-work, behavioral bonus of male leaders’ family involvement. In doing so, we extend research on fatherhood bonuses to include a new manifestation beyond pay and promotions (Glauber, 2008; Hodges & Budig, 2010; Lundberg & Rose, 2002)—but one which can also contribute indirectly to pay and promotion bonuses: follower interpersonal helping. Second, while workers’ families have been highlighted as a key source of their own work motivation (e.g., Howe & Menges, 2024; Menges et al., 2017), we extend this idea to theorize that perceptions of leaders’ family experiences outside of work can also motivate others at work, namely, their followers. That is, we theorize that followers react with a desire to help high WFC male leaders to reduce their own cognitive dissonance, because male leaders’ WFC is stereotype inconsistent. Our third contribution lies in applying moral theory to these phenomena: High WFC male leaders tend to be seen as “victims” of work-life imbalance and worthy of assistance, yet high WFC female leaders are viewed at best, as having misplaced priorities, and, at worst, as bad women; this has implications for “double standards” and related types of gender discrimination at work (Benard et al., 2008). Our focus on dissonance as an explanatory mechanism also aligns with moral theorizing, because it reflects a moral tension originating from cognitive and behavioral inconsistencies in morally challenging situations (Te Brake & Nauta, 2022). Finally, we make a fourth contribution enriching theorizing on the ‘work-family narrative’ (Padavic, et al., 2020), that is, that demands between work and family slows women’s (but not men’s) promotion as a widely accepted explanation for leadership gender inequalities in modern organizations. By integrating leadership and followership research, we highlight an informal dynamic within leader–follower dyads that contributes to the ‘invisibility’ of men’s family involvement in organizations: men’s actual WFC is occluded from leaders and the organizations that employ them via follower helping. That is, because followers exert extra effort to help male (vs. female) leaders who experience high WFC, men’s WFC and its potential impact on their work is reduced or even reversed. Thus, this research also offers practical contributions by improving our understanding of and ability to improve gendered work-life and leadership inequalities.

Taken together, we theorize that male (vs. female) leaders with more perceived WFC generate more interpersonal helping (i.e., helping acts and organizational citizenship behaviors [OCB]) from their followers via follower cognitive dissonance and follower prosocial motivation toward leaders, a dynamic which contributes to the ethical concerns about the persisting inequality between women and men leaders in organizations. We test these ideas by triangulating results across three mixed methods studies: a multi-source field study of leader–follower dyads (*N*_total_ = 290; Study 1) and two, pre-registered, behavioral vignette and recall experiments (*N*_total_ = 920; Studies 2–3), respectively.

## Theory and Hypothesis Development: Follower Reactions to Male Leaders’ WFC via Cognitive Dissonance & Prosocial Motivation

One of the most salient social relations in organizations is the leader–follower relationship (see Martin et al., 2016, 2019, for reviews). As such, followers’ perceptions of leaders play an important role in followers’ reactions toward leaders (Campbell et al., 2008). Status differences between leaders and followers “*provide the contextual backdrop…for interpersonal perception and behavior*” (Campbell et al., 2008, p. 16). For example, leaders’ higher status means they are listened to more than peers at the same level in organizations (Ridgeway & Walker, 1995).

Leaders also shape followers’ understandings of norms and strategies for work-family management (Kossek et al., 2018b; ten Brummelhuis & Bakker, 2012); they often become role models or reference points by bridging work and family domains in concrete, observable ways, such as discussing their family at work (Cheung & Halpern, 2010), displaying photos in their workspace (Hardin et al., 2020), or what has more recently become relevant during the hybrid working era: (unintentionally) featuring children in video or phone calls.^3^ Thus, we take an upward perspective by examining follower perceptions of and reactions to leaders’ WFC.

A critical question in the context of follower responses to leaders that we address by integrating cognitive dissonance (i.e., a state of psychological discomfort due to simultaneously held, relevant, yet conflicting elements of knowledge; Festinger, 1957; Harmon-Jones & Harmon-Jones, 2007) with motivational theories (Batson et al., 2008; Grant, 2007, 2008) is: What drives followers’ supportive responses to leaders’ WFC? To answer this question, we build a theoretical model that follower cognitive dissonance drives behavioral responses (i.e., interpersonal helping) toward leaders by increasing follower prosocial motivation, especially towards male leaders. We focus our theorizing on episodic WFC, because followers ‘rise to the occasion’ to help their leaders in moments of need (e.g., to leave work and attend a child’s sports event or school play; see French et al., 2022). In contrast, if perceived WFC is chronic—as often assessed in work-family research due to traditional survey methodology assessing general WFC levels over time—this can become depleting rather than motivating for followers (Maertz et al., 2019). With chronic perceived leader WFC, the regular and repeated nature of the conflict and apparent need for follower interpersonal help becomes less unusual and more routine, becoming less salient and/or draining followers over time (De Cremer et al., 2009; Hannah et al., 2009). Arguing via the counterfactual, because a greater sense of power decreases perspective taking and helping (Sun et al., 2021), followers who are lower in power than their leaders are more inclined to take the leader’s perspective *and* help leaders when they are perceived to need help.

Critically, we theorize that followers respond with more cognitive dissonance to male (vs. female) leaders’ perceived WFC. Although men and women experience similar levels of WFC (see Shockley et al., 2017, for a review), we focus on followers’ *perceptions* of men’s and women’s WFC—a process that is shaped by gender stereotypes. Specifically, male leaders’ WFC is inconsistent with gender stereotypes that more closely associate women with communality and family, and men with agency and work (Cuddy et al., 2004; Eagly & Wood, 2012; Heilman, 2012). This may be especially true for leaders (Eagly & Karau, 2002) in white collar professional service firms (Padavic et al., 2020). Indeed, male leaders’ WFC is also inconsistent with the work-family narrative: the idea that women’s lack of advancement in organizations is explained by their ostensibly greater conflict between family obligations and the necessarily long hours on the job. In other words, men with high WFC threaten the veracity of the work-family narrative (Padavic et al., 2020), because it means that women are not the only ones experiencing WFC. Thus, for these two reasons—both grounded in gendered stereotypes surrounding WFC—we theorize that male leaders’ perceived WFC generates cognitive dissonance in their followers.

Cognitive dissonance research focuses on individuals’ inconsistencies between their own beliefs and actions (e.g., knowing that smoking is bad for you but still doing it; Johnson, 1968; McMaster & Lee, 1991). However, it can also include inconsistencies in others (Davis & Jones, 1960). Cognitive dissonance triggered by discrepancies in the self or perceived in others both motivate behavior change (see Stone & Fernandez, 2008, for a review), which can be more attainable than changing deeply held beliefs about women and men in organizations (Eagly et al., 2020; Heilman, 2012), expressed in prosocial motivation. The experience of dissonance can reflect a moral tension (Te Brake & Nauta, 2022), in our case, when followers make judgments about leaders’ WFC based on perceptions of the extent to which the leader’s behavior aligns with gendered norms of responsibility and care. Here, we integrate these ideas by theorizing that perceived discrepancies in perceptions of leaders trigger cognitive dissonance in followers. After all, a male leader with high WFC contradicts the widely held stereotypes of gender and family (Cuddy et al., 2004). It conflicts with ideas of ‘ideal workers’ and prototypical leaders (Eagly & Wood, 2012; Gloor et al., 2020; Heilman, 2012; Reid, 2015), while also opposing the widely held and generally unquestioned ‘work-family narrative’ (Padavic et al., 2020). To more precisely explain how these perceived discrepancies affect follower behavior, we integrate motivation—in particular, prosocial motivation—which is inherently interpersonal.

As an other-focused psychological process, prosocial motivation directs employees’ attention towards others’ perspectives, including what might be useful for others (Grant & Berry, 2011). Prosocial motivation refers to the desire to expend discretionary effort based on a concern for helping or contributing to another person’s aims and needs satisfaction (Grant, 2007). For followers, this psychological state wherein they are focused on the goal of benefiting others can be oriented towards their leaders. While prosocial motivation can be driven by a range of motivational states, including egoistic motives (e.g., to gain rewards and avoid punishment) and altruistic motives (e.g., empathetic concern; Batson, 1987; Batson et al., 2008), we focus here on a broadly construed motivation to promote the welfare of the target (Brief & Motowidlo, 1986)—male leaders—triggered by followers’ cognitive dissonance. This idea is also supported by theory (e.g., Grant, 2007) and a longstanding research tradition (e.g., Aknin et al., 2013; Bolino & Grant, 2016; Grant & Shandell, 2022) that one’s perceived impact on the (specific) beneficiaries motivates individuals to make a prosocial difference. In our case, because followers anticipate that their actions can make a difference and help reduce the leader’s WFC, we believe that they are an impactful mechanism to reduce cognitive dissonance.

In sum, the nature of the upward relationship and empathic concern for leaders make followers notice their leaders’ WFC; and cognitive dissonance underlies the motivation of followers to focus on male leaders’ high WFC. Thus, followers who perceive their leaders are experiencing higher (vs. lower) WFC are more prosocially motivated toward those leaders—effects that are moderated by leader gender. Formally:

### Hypotheses 1a-b

Perceived leader WFC positively predicts more follower (a) cognitive dissonance and (b) prosocial motivation toward male (vs. female) leaders.

### Follower Motivation and Interpersonal Help Toward Leaders

In the wake of a perceived cognitive inconsistency, individuals feel tension or discomfort which motivates them toward change to reduce the discomfort—often via action (Festinger, 1957). Changing one’s actions can appear more feasible than changing often deeply held beliefs about women and men in organizations (e.g., their WFC; Padavic et al., 2020). This is illustrated by the fact that gendered stereotypes of communality as a female trait have strengthened over time (see meta-analysis by Eagly et al., 2020) and the hegemonic narrative of WFC as predominantly female is strong, embedded at multiple levels of organizations, and extremely resistant to contradicting evidence (see Padavic et al., 2020). Germane to the current research on gender and follower responses to perceived leader WFC, we propose that there are two relevant norms: societal gender stereotypes *and* the work-family narrative. Thus, followers are motivated to direct their actions toward the target person displaying the inconsistency—in this case, male leaders—in ways that can reduce the inconsistency. Thus, we theorize that even if it does not actually reduce leaders’ WFC, followers’ interpersonal helping behaviors in response to the initial, perceived discrepancy of male leaders’ high WFC reduces dissonance by attenuating the initial discrepancy (Martinie & Fointiat, 2006; Simon et al., 1995) in ways that reduce followers’ cognitive dissonance and discomfort.

Observations that prompt gender role stereotype activation—such as surprise when a father noticeably engages in family roles compared to a mother enacting the same behavior—prompt self-regulatory challenges (Devine, 1989). Even awareness without belief in these gender stereotypes can prompt automatic stereotype activation (Amodio et al., 2007), because this type of conflict monitoring and simultaneous self-regulation can occur unconsciously and automatically (Berns et al., 1997). When conflict—or cognitive dissonance (“I shouldn’t be thinking this way”)—happens, a more deliberate regulatory process is initiated to try to eliminate this unpleasant state. And, critical to our theorizing, self-regulation likely manifests as behavioral reparations for the cognitive act of stereotyping (Amodio et al., 2007)—specifically pro‐social reparative strategies (Kenworthy et al., 2011)—to restore one’s sense of self as a good person.

Decades of research on employee motivation toward extra-role behavior (e.g., Graham, 1989; Organ, 1988; Smith et al., 1983) touts helping as a valuable behavior for leaders and organizations. Conceptualized as giving assistance to another person on an organizationally relevant problem or task at work (Dalal & Sheng, 2019), helping is often included in the broader construct of OCB. More specifically, helping is a form of affiliative OCB (van Dyne & Lepine, 1998) directed towards an individual person (i.e., OCB-I [Individual]; Carpenter et al., 2014). Because employees can help others at work in multiple ways, we conceptualize this construct in two, related ways: as ‘interpersonal helping’ (helping acts and OCB)—both exerted toward a specific referent: the leader. This operationalization aligns with prosocially motivated behavior, “voluntarily helping others with work-related tasks and problems” (King et al., 2005, p. 586).

Critically, the interpersonal helping behavior about which we build theory and examine here is targeted toward leaders. Decades of research have shown that prosocial motivation in its general form is associated with personal initiative and OCB (e.g., helping colleagues; De Dreu & Nauta, 2009; see Bolino & Grant, 2016). However, less research has examined upward influence processes and more targeted forms of prosocial motivation and helping. A notable exception is van Dijke et al. (2018): Although our theorized contextual influence differs from theirs (i.e., procedural justice), their work underscores the idea that followers may channel prosocial motivation via a means through which they can exert their autonomy at work, namely, by going above and beyond for their leaders. We propose that prosocially motivated followers aim to help male leaders when they perceive a cognitive discrepancy between male leader stereotypes and WFC, and this takes the form of upward-directed discretionary work effort to help leaders. At the same time, theories of morality in social relationships, particularly moral typecasting, can also explain these gender differences in leader helping. The motherhood penalty predicts sanctions for women high in WFC but a fatherhood premium for men. In this way, high WFC male leaders are cast as “victims” of work-family balance and therefore blameless in their plight. This relates to help-giving in that “people readily proffer help to a person who appears to be a victim of circumstances but not to an individual who is responsible [here, gendered expectations of women] for his or her own plight” (Weiner, 1980, in Gray & Wegner, 2009, p. 507). Hence, cognitive, moral biases underly the selective help followers provide.

In sum, we theorize that followers’ prosocial motivation explains the relation between leader WFC and interpersonal helping (i.e., help and OCB) toward leaders—patterns that are stronger for male (vs. female) leaders. While followers may still be motivated to help their female leaders for the reasons we mentioned (i.e., leaders’ higher status, the salience of leader–follower social relations), we theorize that followers feel stronger feelings of cognitive dissonance and prosocial motivation toward male leaders with higher perceived WFC.

#### Hypotheses 2a-d

(a) Perceived leader WFC positively predicts followers’ interpersonal helping (i.e., [a] helping acts and [b] OCB) towards male (vs. female) leaders, mediated by follower (c) cognitive dissonance and (d) prosocial motivation.

**Fig. 1:**
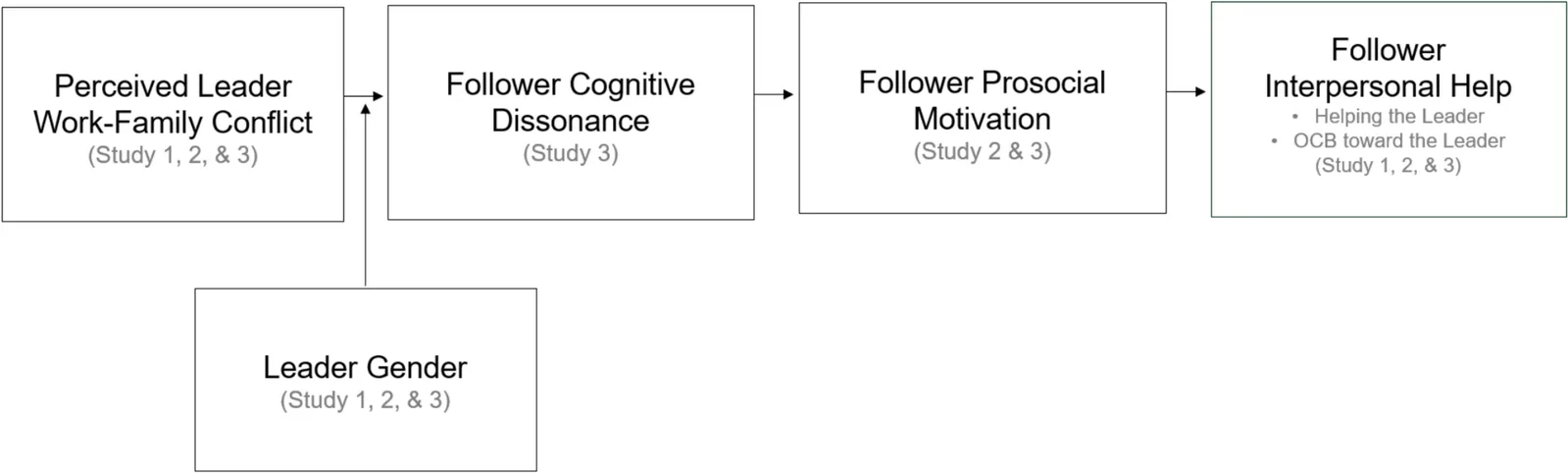
Complete conceptual model

To test our proposed first-stage, serial mediation model (see Fig. 1 for an overview of our complete model), we conducted three studies of leader gender and perceived WFC to triangulate our results (Turner et al., 2017) across methods based on the principles of a full-cycle research approach (Chatman & Flynn, 2005): A field survey to show the effects ‘in the wild’ (Study 1) followed by experiments to show the causal mechanism (Studies 2–3). Ethics approved Study 2 (Studies 1 and 3 were exempt); see OSF^4^ for our data, pre-registrations, and materials.

## Study 1

In a multi-source study of leader–follower dyads in the field, we test follower perceptions of their leaders’ WFC, if those perceptions differ according to leader gender, and how these perceptions predict leader reports of followers’ interpersonal helping.

### Method

#### Sample and Procedure

We collected dyadic data from employed adults in The Netherlands via FlyCatcher, an online, verified panel data service. FlyCatcher contacted participants with leadership responsibility who were interested in participating in a study with an employee. FlyCatcher then invited one of the leader’s direct reports to participate using the contact information the leader provided (see Online Appendix for more details) and compensated both persons for quality, complete responses.

From the 268 leaders and followers who started the survey, our final data included 145 dyads (54.0% completion rate, which is only slightly lower than the typical response rates for dyadic studies; see Holtom et al., 2022). Of these 145 leaders, 77.9% identified as women and 22.1% as men. Almost half (43.8%) of the leaders had at least one older child (i.e., > 10 years), 27.1% had at least one young child (i.e., < 10 years), and 29.2% did not have children; almost all (93.8%) leaders had a partner *or* children. Of the 145 followers, 65.5% identified as women and 34.5% as men. A quarter (25.7%) of followers had at least one older child (i.e., > 10 years), 30.6% had at least one young child (i.e., < 10 years), and 43.8% were childfree. Leaders and followers had worked together for an average of 8.05 years (*SD* = 6.80). We did not ask about industry due to space constraints; however, according to FlyCatcher, the participants in the panel hailed from industries such as real estate, finance, consultancy, health, and public administration.

### Measures

For this and future studies, all items were presented in a random order within each scale and measured from (1) “Strongly Disagree” to (5 or 7) “Strongly Agree” (unless noted). We forward- and back-translated Study 1 measures with native speaker social science experts, in accordance with best practices. For the complete scales, CFAs, and model fit, see the Online Appendix.

#### Perceived Leader WFC

We used three items from Carlson et al. (2000) to assess followers’ perceptions of their leaders’ WFC,^5^for example, “my leader has to miss family activities due to the amount of time they must spend on work responsibilities” (*α* = .85, Ω = .86), which we standardized in the analyses to facilitate interpretation of the results.

#### Interpersonal Helping Toward Leaders

We also used two scales to assess followers’ interpersonal helping toward leaders, as rated by leaders to avoid socially desirable responding. First, we adapted a 4-item measure of helping the leader from a general measure of helping acts by Gabriel et al. (2018) and Glomb et al. (2011). Items included, “*My follower helped me with tasks outside of the scope of work*.” Items were rated with the same anchors as the previous scales, but from 1 to 5 (*α* = .78, Ω = .79). Second, we used a 6-item measure of OCB toward supervisors (Cropanzano et al., 2003a, 2003b) to examine followers’ OCB directed toward the leader. The items assessed actions directed toward one’s leader and are predominantly helping behaviors (e.g., “*help me with some of my work even if not asked*;” *α* = .88, Ω = .85). Thus, we consider it a second measure of interpersonal helping toward leaders, but we refer to it as OCB, for clarity.

#### Covariates

Follower gender may be relevant (Vial et al., 2018). But it did not predict (*b* = − 0.07, *SE* = .03, *z* = − 1.23, *p* = .220), nor moderate our main results (*b* = − 0.28, *SE* = .20, *z* = − 1.37, *p* = .172), replicating other multi-source leader–follower studies (e.g., van Gils et al., 2018). Furthermore, we assessed overall prosocial motivation toward the leader, given its relevance for our model and testing in subsequent studies. To do so, we used a one-item, adapted measure of general prosocial motivation (Grant, 2008) that leaders reported about their followers: “how motivated is your follower to support you?” (1 “not at all motivated” to 7 = “very motivated”).^6^

**Table 1:** Descriptive statistics and correlations (Study 1)

Variables	*M*	*SD*	1	2	3	4	5
1. Leader gender	0.22	0.42	–				
2. Leader WFC	2.52	1.00	− 0.01	–			
3. Helping the leader	3.51	0.71	0.14	− 0.13	–		
4. OCB toward leader	3.78	0.63	0.12	− 0.17*	0.79***	–	
5. Follower gender	0.34	0.48	0.17*	0.03	− 0.01	0.07	–
6. General prosocial motivation	5.58	0.94	0.03	− 0.11	0.45***	0.57***	0.05

Leader gender was coded female (0) and male (1), follower gender was coded female (0), male (1). There were 145 complete dyads from 145 followers and leaders (thus, our data reflect a total *N* = 290)

*WFC* Work-family conflict

*p < .05; **p < .01; ***p < .001

### Results

We used path analysis with GSEM (Stata 18.0) with robust *SE*s. We calculated the direct and interactive effects of follower perceptions of leader WFC, leader gender, and follower interpersonal helping (i.e., followers’ help and OCB toward the leader). However, our CFAs show helping and OCB were not empirically distinct (see Online Appendix). Thus, the results for helping may arguably be the most valid because it was asked before OCB in the survey; still, we present the results for both for transparency. For descriptives and correlations, see Table 1.

**Fig. 2:**
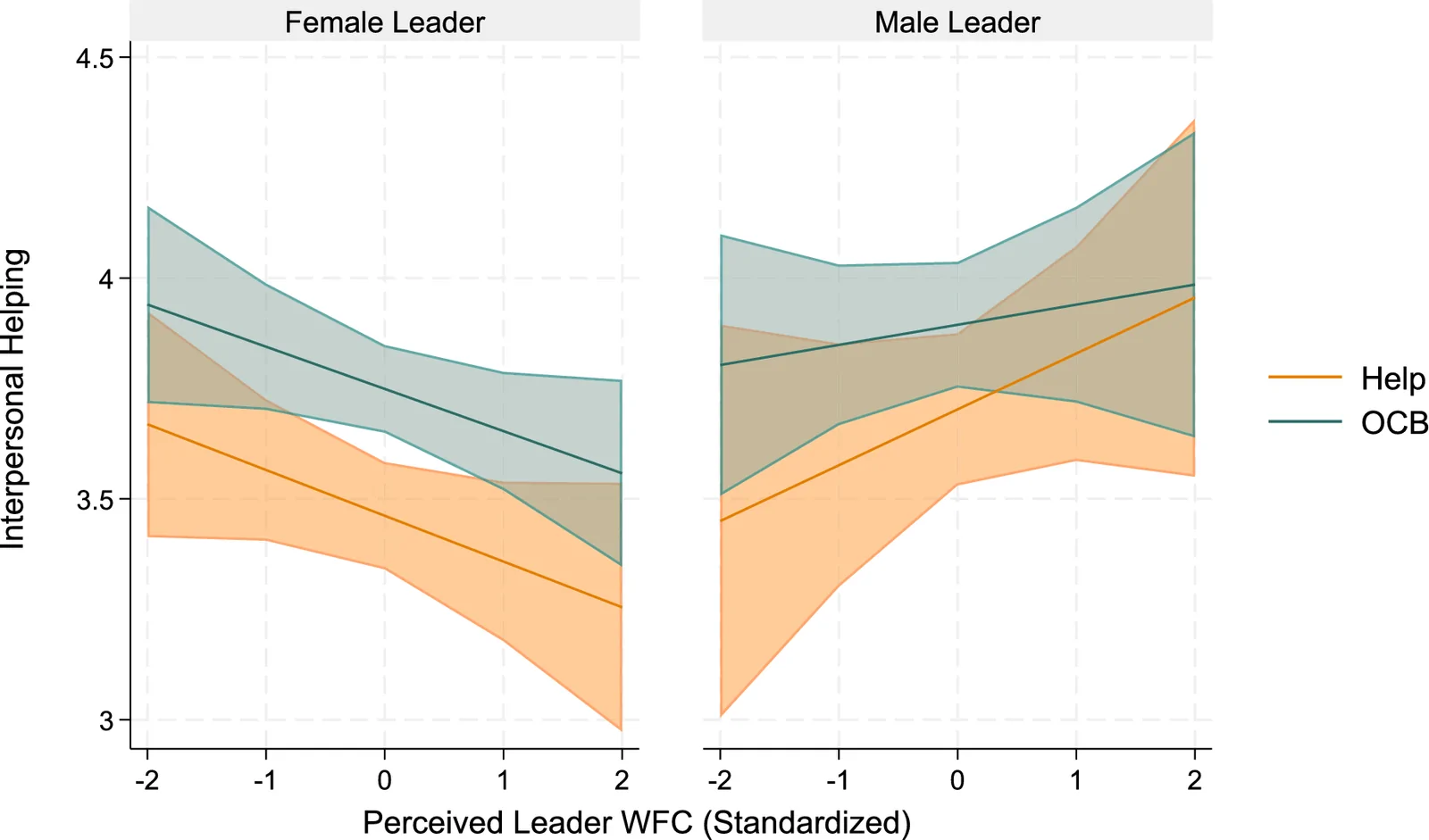
Helping the leader by leader WFC and gender (Study 1). Error bars reflect 95% CIs. Data are dyadic from 145 followers and 145 leaders (total *N* = 290)

Perceived leader WFC^7^ interacted with leader gender to predict helping leaders, *b* = 0.23, *SE* = .12, *z* = 1.99, *p* = .047, such that perceived leader WFC increased helping toward male leaders (*simple slope* = .13, *SE* = .10, *z* = 1.27, *p* = .203) but decreased helping toward female leaders (*simple slope* = − 0.10, *SE* = .06, *z* = − 1.69, *p* = .091; see Table 2; Fig. 2), which did not significantly differ from zero but significantly differed from each other. These results revealed a more positive effect of perceived leader WFC on helping for male (vs. female) leaders, supporting H2a.

**Table 2:** Path analysis results (Study 1)

Variable	Helping the leader		OCB toward leader	
	*b *(*SE*_robust_)	*b* (*SE*_robust_)	*b* (*SE*_robust_)	*b* (*SE*_robust_)
Constant	1.76 (0.38)***	1.88 (0.39)***	1.80 (0.37)***	1.88 (0.37)***
Leader gender	0.24 (0.11)*	− 0.34 (0.32)	0.14 (0.09)	− 0.21 (0.23)
Leader WFC	− 0.06 (0.05)	− 0.10 (0.06)	− 0.07 (0.04)	− 0.10 (0.05)
Leader gender × Leader WFC	–	0.23 (0.12)*	–	0.14 (0.09)
Follower gender	− 0.08 (0.12)	− 0.07 (0.12)	0.04 (0.09)	0.04 (0.09)
General prosocial motivtation	0.33 (0.06)***	0.33 (0.06)***	0.37 (0.05)***	0.38 (0.05)***

Leader gender coded female (0), male (1). There were 145 complete dyads from 145 followers and leaders (thus, our data reflect a total *N* = 290)

*WFC* Work-family conflict

^*^p < .05; **p < .01; ***p < .001

Perceived leader WFC did not significantly interact with leader gender to predict OCB toward leaders, *b* = 0.14, *SE* = .09, *z* = 1.61, *p* = .106, such that perceived leader WFC increased OCB toward male leaders (*simple slope* = .05, *SE* = .07, *z* = 0.62, *p* = .537) but decreased OCB toward female leaders (*simple slope* = − 0.09, *SE* = .05, *z* = − 1.94, *p* = .053; see Table 2; Fig. 2)—neither achieving acceptable levels of statistical significance from zero nor from each other. These results revealed trending patterns of a more positive effect of perceived leader WFC on OCB for male (vs. female) leaders, echoing the previously reported pattern of results for helping; however, given the levels of significance, we cannot definitively say these results support H2b.

By showing more pronounced follower help towards male (vs. female) leaders with higher perceived WFC in the field, these results provide initial support for our theorizing.

## Study 2

We have three key aims in this study: First, we test the mediator that male (vs. female) leaders with higher WFC generate more follower helping via episodic prosocial motivation toward the leader. Second, by manipulating leader WFC *and* gender, we can causally measure participants’ reactions towards male and female leaders with high and low WFC. Third, we test tangible helping outcomes via objective, discretionary behaviors toward leaders to replicate our effects using actual rather than reported behaviors (as in Study 1). These behaviors (e.g., providing feedback on a job advertisement for a leader) are appropriate indicators of participants’ prosocially motivated helping acts toward leaders because the actions require participants’ time but do not increase their compensation. Together, these design choices strengthen the internal and external validity of our results (Highhouse, 2009; Podsakoff & Podsakoff, 2019; Turner et al., 2017).

## Study 2 Method

### Sample and Procedure

We recruited employed German adults via Prolific Academic. In total, 502 participants began our survey, and 450 participants provided complete data on our key measures. As outlined in our pre-registration, we then excluded those who responded incorrectly to two or three of the objective manipulation and comprehension checks (*n* = 30) for a final sample of 420, exceeding our pre-registered goal of 400. Of these 420 participants, 58.1% identified as men and 41.9% as women and 11.0% had children. On average, participants were 28.36 years old (*SD* = 8.41) with 6.04 years (*SD* = 7.36) of work experience and working in various industries such as academia/education (22.14%), technology (20.71%), retail/service (8.33%), healthcare (5.95%), marketing/communication (5.24%), finance/accounting (5.24%), and other (32.39%). All were paid 1.01GBP.

Participants were asked to take the role of a follower and randomly assigned to view 1 of the 4 conditions in a between-subjects design (leader WFC: high or low, leader gender: male or female). The stimuli included statistics about work and family time, the leader’s comments on work and life (e.g., “I have to work [so much]… which makes it difficult to balance work and life as I would like to.”). Leader gender was indicated with two different names (e.g., “Jan” or “Christian” for men, “Stefanie” or “Julia” for women—common names in Germany for today’s leaders) and pronouns (e.g., “he” and “she”); we used multiple names to ensure our results were not artifacts associated with one particular stimulus and indicative of the broader patterns of results, enhancing internal and external validity with a stimulus sampling approach (Wells & Windschitl, 1999). All leaders were described as having a partner and children. By focusing on higher level leaders working in white-collar, professional organizations with long work hours, we approximate a prototypical employment context where work-family narratives are strongest: in white collar, high-status firms (see Padavic et al., 2020). See the Online Appendix for the full text.

### Measures

#### Prosocial Motivation Toward Leaders

We adapted a 5-item measure of employees’ general prosocial motivation by Grant (2008) to measure participants’ episodic prosocial motivation towards the leader. We asked, “how motivated are you to support the leader?” (1 “not at all motivated” to 7 = “very motivated”), followed by the stem, “…Why were you motivated to support the leader?” and four items including, “Because I wanted to help the leader with my work.” We forward- and back-translated the items with native German speakers (Ω = .86).

#### Helping Leaders

We measured four objective, behavioral outcomes to indicate participants’ help toward leaders. We told participants that the leader they just read about was looking for people to join their team; we asked participants if they were willing to read the job advertisement (coded as yes [1] or no [0]) and if they would provide feedback on it (i.e., how to make the advertisement better and/or more attractive) with an open-ended text field—the first behavioral outcome. This text field was coded as yes (1) or no (0) if the participant added text—the second behavioral outcome. Examples of participant feedback about the job advertisement included: “Too much text, too little advantage for an applicant (it only sounds like a lot of work and there is no real selling point)” and “Shorten key points, make them more concise and create advantages that [the company] offers as an employer (bonuses, teamwork, etc.).” We also included the number of characters in the feedback (log-transformed) in our summative, 3-item indicator of help (Ω = .88). See below for more information about our fourth indicator, which was (too) strongly correlated with this third indicator (i.e., *r* = .99, *p* < .001), and thus dropped.

#### Control Variables

We included self-reported participant gender: male (1) or female (0), but our results did not differ for male and female participants (*p* = .403) and were thus collapsed. We also included a variable for the stimuli version to which participants were randomly assigned (e.g., 0 or 1 for Jan/Julia or Christian/Stefanie). Finally, as mentioned in our pre-registration, we also asked participants about the acceptability of overtime hours (i.e., “working overtime hours are…completely unacceptable [1]” to “completely acceptable [5]”), which may account somewhat for the team culture and/or industry in which participants work, influencing leaders’ WFC and followers’ ability, motivation, and/or willingness to help them.

## Study 2 Results

### Analysis Plan

We analyzed data as in Study 1. Descriptives and correlations are reported in Table 3.

**Table 3:** Descriptive statistics and correlations (Study 2)

Variables	*M*	*SD*	1	2	3	4	5
1. Leader gender	0.50	0.50	–				
2. Leader WFC	0.60	0.49	0.01	–			
3. Episodic prosocial motivation	4.39	1.30	− 0.002	0.11*	–		
4. Helping the leader	3.16	2.88	− 0.09	0.02	0.16***	–	
5. Follower gender	0.58	0.49	0.03	− 0.03	− 0.08	− 0.03	–
6. Stimuli version	0.50	0.50	− 0.05	0.01	0.10*	0.07	0.04

Leader gender coded female (0) and male (1). WFC coded low (0) and high (1). Participant gender coded female (0) and male (1). *N* = 420

*WFC* Work-family conflict

*p < .05, **p < .01, ***p < .001

### Main Analyses

**Fig. 3:**
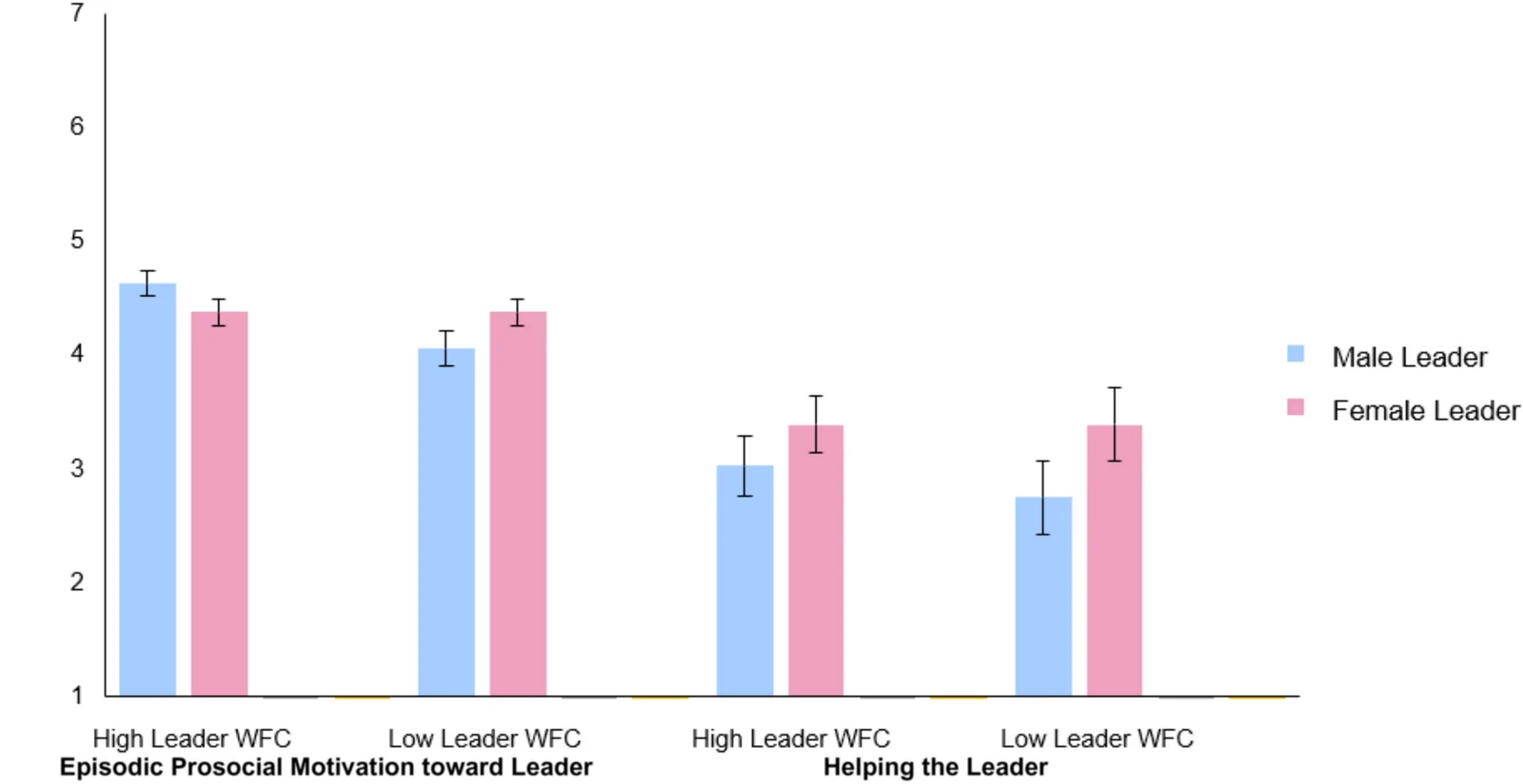
Effects of leader gender and WFC (Study 2). *WFC* Work-family conflict. Error bars reflect *SE*s. *N* = 420

Leader WFC predicted more episodic prosocial motivation towards the leader (*M*_lowwfc_ = 4.22, *SD* = 1.31; *M*_highwfc_ = 4.51, *SD* = 1.28, *d* = .23; *b* = 0.28, *SE*_robust_ = 0.13, *p* = .027; see Table 4; Fig. 3). Leader gender did not predict episodic prosocial motivation toward the leader (*b* = 0.01, *SE*_robust_ = 0.13, *p* = .928). But as hypothesized, these effects were qualified by a significant interaction (*b* = 0.57, *SE*_robust_ = 0.26, *p* = .027), such that with high (vs. low) leader WFC, participants reported more prosocial motivation toward male (*simple slope:* 0.57, *SE* = 0.19, *p* = .003) than female (*simple slope*: 0.001, *SE* = 0.17, *p* = .994; see Fig. 3) leaders. These results causally replicate Study 1, supporting Hypothesis 1b, which hypothesized leader WFC predicts more follower episodic prosocial motivation toward male (vs. female) leaders.

**Table 4:** Moderated mediation model (Study 2)

Variable	Episodic prosocial motivation		Helping the leader	
	*b* (*SE*_robust_)	*b* (*SE*_robust_)	*b* (*SE*_robust_)	*b* (*SE*_robust_)
Constant	4.20 (0.15)***	4.37 (0.16)***	3.27 (0.40)***	1.76 (0.61)**
Leader gender	0.01 (0.13)	− 0.33 (0.20)	− 0.64 (0.45)	− 0.53 (0.44)
Leader WFC	0.28 (0.13)*	0.00 (0.17)	− 0.002 (0.41)	− 0.00 (0.40)
Leader Gender × leader WFC	–	0.57 (0.26)*	0.28 (0.57)	0.09 (0.57)
Participant gender	− 0.19 (0.13)	− 0.20 (0.13)	− 0.13 (0.28)	− 0.06 (0.28)
Stimulus	0.24 (0.13)	0.25 (0.12)*	0.40 (0.28)	0.31 (0.28)
Episodic prosocial motivation	–	–	–	0.35 (0.11)***

Low (0), high (1). Leader gender and participant gender coded male (1), female (0) *N* = 420

*WFC* Work-family conflict

*p < .05, **p < .01, ***p < .001

As predicted, the indirect effect calculated with 20,000 bootstrapped resamples and bias-corrected CIs (MacKinnon et al., 2004) showed that the indirect effect of leader WFC on helping the leader via episodic prosocial motivation was positive and differed from zero for male leaders (0.20, *SE*_boot_ = .09, 95% CI_bc_ [0.06, 0.43]); however, it did not significantly differ from zero for female leaders (0.00, *SE*_boot_ = .06, 95% CI_bc_ [− 0.12, 0.13]); these two values significantly differed (.20, *SE*_boot_ = .11, 95% CI_bc_ [.03, .48]). These results support H2a and H2d: leader WFC predicted more follower interpersonal helping towards male (vs. female) leaders, explained by episodic prosocial motivation.

## Study 3

In Study 2, episodic prosocial motivation toward the leader explained the gendered process of how male—but not female—leaders’ perceived WFC generates more follower helping. However, there are additional challenges to assess cognitive dissonance in survey research (i.e., merely asking about an uncomfortable emotional state like dissonance can alleviate it and/or its downstream effects; Double & Birney, 2019; Kassam & Mendes, 2013). Hence, to try show explicit evidence of this mediator and a key part of our theoretical model, we explicitly measure and test cognitive dissonance as a mediator, such that male (vs. female) leaders with higher WFC generate more helping from their followers via cognitive dissonance and prosocial motivation. Using an episodic design as in Study 2, we further complement Study 2’s vignette design with an experimental recall study which maximizes the internal and the external validity of the design as real situations with randomization to recall different kinds of real situations, while also triangulating our causal results across methods and measures (Turner et al., 2017). By using a neutral comparison condition (i.e., “small talk”) instead of a levels comparison condition (i.e., low vs. high WFC in Study 2), we also conservatively test our ideas with a robust baseline for comparison ensuring the effects we are observing are driven by high—not low—perceived WFC.

## Study 3 Method

### Sample and Procedure

We recruited adults with direct supervisors in the United Kingdom via Prolific Academic. After 641 participants began our initial screening survey, we invited 586 eligible participants (i.e., currently employed and working in white collar industries) to the main survey and 89.8% provided complete data. As pre-registered, we excluded those who did not follow instructions (i.e., did not write a coherent story or responded incorrectly to the attention check, “Please click ‘strongly disagree’,” which was nested among the other items; *n* = 25). Of the final 500 participants, 53.6% identified as men, 45.8% as women, and 0.01% as other. Half (52.2%) reported having children. Participants worked an average of 4.87 years (*SD* = 4.96) with their leaders. They hailed from healthcare (17.8%), education (14.6%), technology (12.0%), engineering (12.0%), finance (8.2%), and professional services (7.6%). All were paid 1.5 GBP.

Most leaders about which participants reported had children (75.8%) and were female (45.6%) or male (54.2%) except one person identifying as other (0.02%). Due to our gendered hypotheses and insufficient subsample size, the non-binary leader was excluded from the model.

We randomly assigned participants to 1 of 2 conditions (i.e., [a] high work-family conflict or [b] a neutral control) in a between-subjects recall experiment. Based on Grube et al.’s (2008) adaptation of the day reconstruction method (Kahneman et al., 2004), which Gloor et al. (2022) modified to recall specific events, we prompted participants to “think of a time when your leader had (a) high work-family conflict, or their work and family domains were incompatible” or “ (b) had a routine interaction with your leader that felt typical or uneventful.” We then provided more description based on our definition of WFC (Greenhaus & Beutell, 1985) and our measure of WFC in Study 1 (Carlson et al., 2000) or the neutral activity of “small talk” (i.e., trivial communication not core to task completion; Methot et al., 2021), respectively. We invited participants to “take some time to write about this instance” focused on their thoughts, feelings, and the surrounding circumstances “with enough detail that someone else could understand the situation.” We required at least 200 characters of written text before participants could advance to the next step of the survey. For the complete text manipulations, see the Online Appendix.

### Measures

#### Cognitive Dissonance

As previously mentioned, cognitive dissonance is difficult to measure via self-report. Thus, as we pre-registered, we assessed cognitive dissonance in four ways via related measures (rated from 1 to 7): psychological discomfort (3 items from Elliot & Devine, 1994; Ω = .95), guilt (2 items from Watson et al. [1988], Spearman-Brown corrected estimate = .87), and a single value indicator of “cognitive dissonance” based on our definition using large language models via the Generative Pre-trained Transformer (GPT 3.5; OpenAI, 2024). Specifically, we prompted GPT to assess participants’ text after the experimental prompt and provide a single score from 1 (“not at all”) to 7 (“very much”) reflecting “the state of psychological discomfort due to simultaneously held, relevant, yet conflicting elements of knowledge.” Finally, we also included “discrepancy” values calculated with LIWC-22 (Boyd et al., 2022), following previous research (e.g., Martinie et al., 2013).^8^

#### Interpersonal Helping

We assessed OCB (Ω = .91) and helping (Ω = .89) as in Study 1.

#### Episodic Prosocial Motivation

We assessed followers' prosocial motivation toward their leaders in the recalled episode as in Study 2 (α = .94).

#### Control Variables

Results for men and women did not significantly differ for any of the cognitive dissonance measures (*ps* = .06–.90) and, thus, we collapsed results across participant gender.

## Study 3 Results

### Analysis Plan

We analyzed our data as in Study 2 using Stata, which is a minor departure from our pre-registration (i.e., lavaan/processR) due to endogeneity, because Stata is more convenient for endogeneity-corrected structural models. We found that endogeneity undermined our serial mediation analysis (Antonakis et al., 2014; see the Online Appendix); however, our instruments for the potentially biased second mediator, prosocial motivation, were weak (*F*[2, 489] = 3.66, *p* = .027). Thus, we focus here on the results calculated without the endogeneity controls which were also robust across different models. However, we also present additional results with endogeneity controls in the Online Appendix for transparency. For descriptives and correlations, see Table 5. For CFAs and model fit statistics, see the Online Appendix.

**Table 5:** Descriptive statistics and correlations (Study 3)

Variables	*M*	*SD*	1	2	3	4	5	6
1. Leader gender	0.54	0.50	–					
2. Leader WFC	0.48	0.50	− 0.04	–				
3. Cognitive dissonance (GPT)	2.68	1.33	0.13**	0.64***	–			
4. Cognitive dissonance (Guilt)	1.91	1.37	0.16***	0.43***	0.78***	–		
5. Episodic prosocial motivation	5.44	1.29	− 0.007	− 0.06	− 0.13**	0.02	–	
6. Helping the leader	5.11	1.38	− 0.01	0.07	0.07	0.13**	0.63***	–
7. OCB toward leader	5.33	1.25	0.001	0.12**	0.11*	0.14**	0.62***	0.80***

Leader gender coded female (0) and male (1). WFC coded neutral/control (0) and high (1). *N* = 500 (some values calculated with *N* = 499, because 1 leader identified as not male nor female)

*WFC* Work-family conflict

*p < .05, **p < .01, ***p < .001

#### Main Analyses

**Fig. 4:**
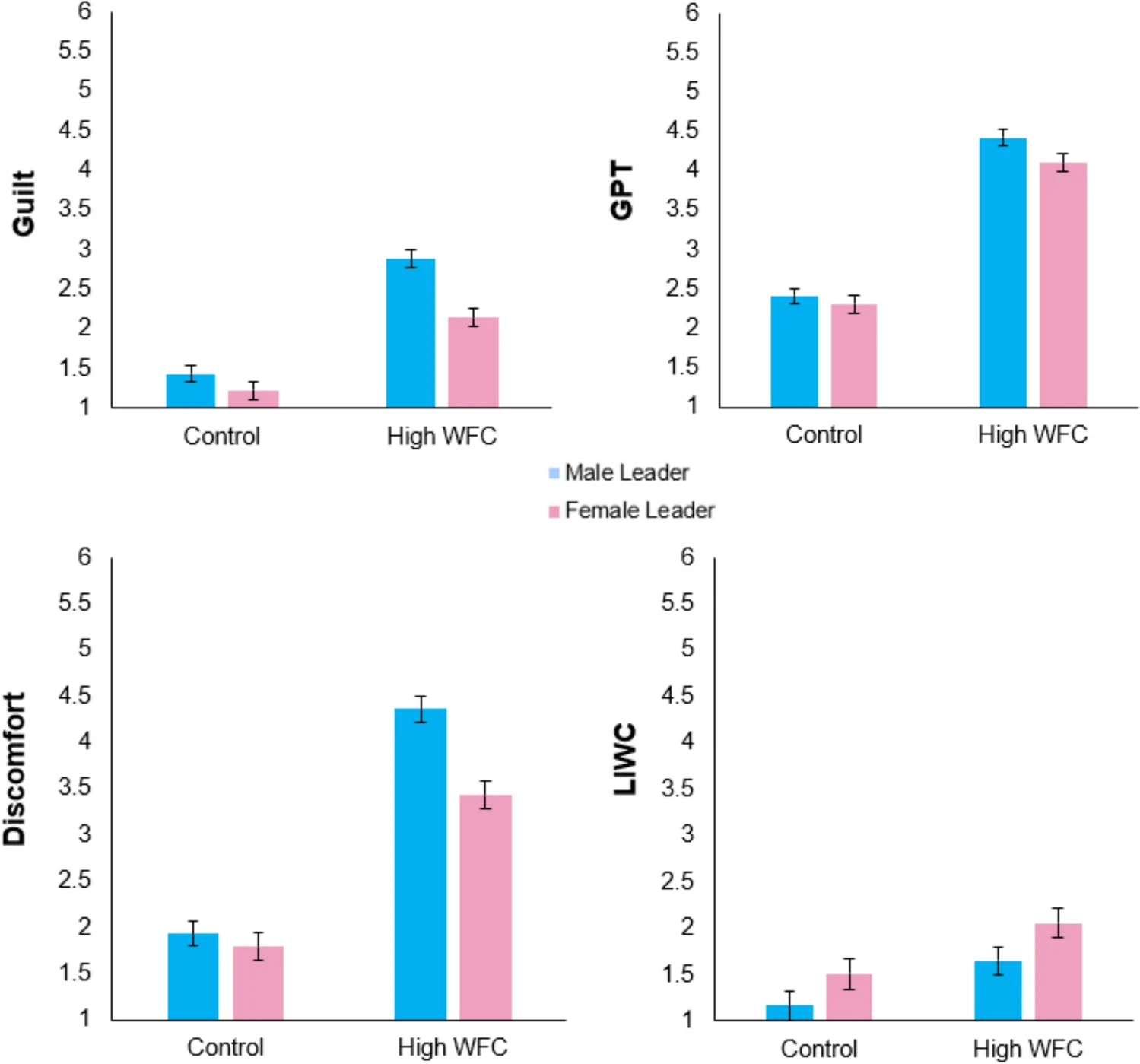
Effects of leader gender and WFC on cognitive dissonance (Study 3). *WFC* Work-family conflict. Error bars reflect *SE*s. *N* = 499, because 1 leader identified as not male nor female

Across all four measures of cognitive dissonance, leader WFC (vs. control) significantly predicted more cognitive dissonance (e.g., for guilt: *M*_control_ = 1.86, *SE* = .05; *M*_highwfc_ = 3.57, *SE* = .07, *d* = 1.66; *t*[498] = 18.57, *p* < .001; see Table 6). Only for guilt did leader gender also significantly predict cognitive dissonance (*M*_male_ = 2.84, *SE* = .09; *M*_female_ = 2.50, *SE* = .08, *d* = .26; *t*[497] = 2.84, *p* = .002). As predicted, these effects were qualified by a significant interaction for all four measures except for LIWC (e.g., for guilt: *b* = 0.53, *SE*_robust_ = 0.22, *z* = 2.43, *p* = .015: high leader WFC (vs. control) generated more cognitive dissonance for male [*simple slope*: 1.44, *SE* = 0.17, *z* = 8.29, *p* < .001] vs. female [*simple slope*: = 0.91, *SE* = 0.13, *z* = 7.03, *p* < .001] leaders, slopes that significantly differed, *χ*^2^(1) = 5.91, *p* = .015, see Fig. 4). These results support H1a, which predicted leader WFC generates more follower cognitive dissonance toward male (vs. female) leaders. Results did not show more prosocial motivation toward male leaders with high WFC (*b* = .03, *SE*_robust_ = .23, *p* = .889), providing no support for H1b (and ostensibly conflicting with Study 2 results); however, we asked prosocial motivation *after* cognitive dissonance in the survey, which—based on our aforementioned explanation—could have dampened these effects.

**Table 6:** Moderated serial mediation model (Study 3)

Variable	CogDiss (GPT)		CogDiss (Guilt)	Episodic prosocial motivation	Helping the leader	OCB toward leader
	*b* (*SE*_robust_)		*b* (*SE*_robust_)	*b* (*SE*_robust_)	*b* (*SE*_robust_)	*b* (*SE*_robust_)
Constant	− 0.68 (0.06)***		− 0.50 (0.05)***	− 0.06 (0.11)	5.14 (0.10)***	5.32 (0.11)***
Leader WFC	1.08 (0.09)***		0.67 (0.09)***	0.12 (0.17)	0.02 (0.16)	0.05 (0.16)
Leader gender	0.11 (0.08)		0.16 (0.07)*	− 0.03 (0.12)	− 0.12 (0.13)	− 0.10 (0.13)
Leader gender × WFC	0.38 (0.13)**		0.38 (0.16)*	0.07 (0.17)	0.09 (0.19)	0.16 (0.17)
CogDiss (GPT)	–		–	− 0.44 (0.11)***	0.17 (0.09)^†^	0.24 (0.08)**
CogDiss (Guilt)	–		–	0.33 (0.09)***	0.03 (0.07)	− 0.06 (0.06)
Episodic prosocial motivation	–		–	–	0.89 (0.06)***	0.81 (0.05)***

Leader gender (female = 0, male = 1), Leader work-family conflict (WFC: control = 0, high = 1). CogDiss = Cognitive Dissonance. CogDiss and Episodic Prosocial Motivation are standardized. *N* = 499, as 1 leader identified as not male/female

^†^*p* = .055, *p < .05, **p < .01, ***p < .001

The complete model with all four measures of cognitive dissonance showed only the GPT measure (*p* = .020) and guilt (*p* = .001) significantly predicted the second mediator: prosocial motivation; discomfort and the LIWC measure were both non-significant predictors (*p*s = .462–.871). Furthermore, neither discomfort nor LIWC consistently and significantly predicted our interpersonal helping outcomes (*p*s = .022–.155 for LIWC; *p*s = .624–.993 for discomfort). Thus, we present the rest of our moderated serial mediation model calculated only with the GPT and guilt measures below; however, we share alternate models in the Online Appendix.

As predicted, the indirect effect calculated with 20,000 bootstrapped resamples and bias-corrected, accelerated CIs showed that for female leaders, leader WFC predicted help (0.19, *SE*_boot_ = 0.06, *z* = 3.13, *p* = .002, 95% CI_bca_ [.07, .31]) and OCB (0.18, *SE*_boot_ = 0.05, *z* = 3.21, *p* = .001, 95% CI_bca_ [.07, .28]) toward the leader via cognitive dissonance (guilt) and episodic prosocial motivation; patterns were stronger for male leaders on help (0.31, *SE*_boot_ = 0.10, *z* = 3.10, *p* = .002, 95%CI_bca_ [.11, .50]) and OCB (0.28, *SE*_boot_ = 0.09, *z* = 3.17, *p* = .002, 95%CI_bca_ [.11, .44]). The indirect effects for men and women leaders differed with marginal significance on help (0.11, *SE*_boot_ = 0.06, *z* = 1.88, *p* = .061, 95%CI_bca_ [− 0.005, 0.23]) and OCB (0.10, *SE*_boot_ = 0.05, *z* = 1.89, *p* = .059, 95%CI_bca_ [− 0.004, .21]).

For the other measure of cognitive dissonance (GPT), the patterns of results were similar except the relation between cognitive dissonance and episodic prosocial motivation was negative, which also changed the indirect effect valence from positive to negative. Specifically, for female leaders, leader WFC predicted help (− 0.42, *SE*_boot_ = 0.11, *z* = − 3.87, *p* < .001, 95% CI_bca_ [− 0.63, − 0.21]) and OCB (− 0.38, *SE*_boot_ = 0.10, *z* = − 4.01, *p* < .001, 95% CI_bca_ [− 0.57, − 0.20]) toward the leader via cognitive dissonance (GPT) and episodic prosocial motivation; results showed stronger patterns for male leaders predicting help (− 0.57, *SE*_boot_ = 0.16, *z* = − 3.64, *p* < .001, 95%CI_bca_ [− 0.88, − 0.26]) and OCB (− 0.52, *SE*_boot_ = 0.14, *z* = − 3.77, *p* < .001, 95%CI_bca_ [− 0.79, − 0.25]). The indirect effects for men and women leaders significantly differed on help (− 0.15, *SE*_boot_ = 0.07, *z* = − 2.06, *p* = .040, 95%CI_bca_ [− 0.29, − 0.007]) and OCB (− 0.14, *SE*_boot_ = 0.06, *z* = − 2.09, *p* = .036, 95%CI_bca_ [− 0.26, − 0.009]).

Critically, none of our core findings consistently and meaningfully changed when calculated with models including the prosocial motivation endogeneity control-function instrument (a variable that did not significantly predict helping nor OCB, *p*s = .263—.464, providing additional evidence beyond our previously reported Wald test that our instruments are weak) or with the inclusion of covariates in the model, suggesting they are generally robust results.

Taken together, our results calculated with cognitive dissonance (guilt)—but not cognitive dissonance (GPT)—support Hypothesis 2c-d: leader WFC generated more follower interpersonal helping towards male (vs. female) leaders, mediated by cognitive dissonance and prosocial motivation. As we discuss more in the Online Appendix, because the GPT measure captured cognitive dissonance as conflict (which *reduced* motivation), but guilt captured a more moral, emotional dissonance a la Te Brake & Nauta (2002; which *increased* motivation), the dissonance indicators operated through different psychological systems, thus producing divergent behavioral effects; only the latter, moral cognitive dissonance, supports our theorizing.

## General Discussion

Do followers’ gendered perceptions of their leaders’ WFC explain leaders’ fatherhood bonuses from follower interpersonal helping? Findings from three mixed methods studies generally supported our theorizing, such that participants engaged in more helping and OCB toward male (vs. female) leaders who were ascribed more WFC, explained by greater (moral) cognitive dissonance and (episodic) prosocial motivation towards these leaders. Now, we highlight these findings’ implications for theory and practice related to gendered leader–follower dynamics at the work-family interface, as well as for business ethics and workplace inequality more broadly.

### Theoretical Implications

First, our results extend existing work on perceived WFC across hierarchies, which has tested leaders’ perceptions of followers’ WFC in the typical (downward) direction of status and power relations in organizational hierarchies (Dosier et al., 1988). Because status and power are particularly defining, pervasive, and influential features at work for lower status individuals (see Magee & Galinsky, 2008), we examined the upward direction of influence (i.e., followers’ perceptions of leaders’ WFC). Our results suggest that this is a key distinction: while downward evaluations have demonstrated negative outcomes for targets with higher WFC (e.g., negative career effects such as lower performance evaluations; Carlson et al., 2008; Hoobler et al., 2009; King, 2008), upward evaluations seem to operate in the opposite fashion—predicting *positive* outcomes for (high-status) targets with higher WFC. Specifically, we found positive behavioral effects in the form of followers helping their male leaders—not just despite their WFC, but seemingly *because* of their WFC. This evidence complements a developing area of research on followership (e.g., Uhl-Bien et al., 2014; van Vugt, 2006), which emphasizes followers as understudied yet active and influential members of the leader–follower dyad. This work also extends current WFC theorizing, supporting broader research on status accumulation and ‘fatherhood bonuses’ (e.g., Berger et al., 1972; Gatrell et al., 2022; Ridgeway & Walker, 1995). Finally, our findings also illuminate the work-family and leadership/followership literatures which have focused on how leaders influence follower WFC (e.g., family-supportive supervision; Crain et al., 2018; Hammer et al., 2011). Instead, we show how and when followers notice and respond to leaders’ WFC in ways that may influence leaders’ actual WFC; that is, these follower perceptions generate more follower effort and resources for the leader on the job.

This research showed positive behavioral effects of perceived WFC by integrating prosocial motivation theory (Grant, 2007, 2008). We argued that lower-level evaluators (e.g., followers) are not only attentive to leaders’ work-family demands and challenges (Kossek et al., 2018a; ten Brummelhuis & Bakker, 2012), but they are also attuned to leaders’ socio-emotional needs—and thus, are motivated to help their superiors. As a result of leaders’ greater ascribed WFC, our findings suggest that participants aimed to fill this apparent need through prosocially-motivated “care” of the leader (Kuvaas et al., 2012; Rynes et al., 2012) or behaviors enacted to improve the leaders’ general welfare (Brief & Motowidlo, 1986), by exerting more interpersonal help toward them. This is a key difference in the effects previous research has shown for followers with high WFC: with negative evaluations and career sanctions, versus how observers of leaders with high WFC are treated in the current research: prosocially and with extra-role behavior. The latter we describe as “prosocial but with possible benefits:” While followers are apparently benevolent toward leaders with high WFC, the underlying thought may be that helping the high WFC leader feeds a high-quality exchange relationship, generating more desirable resources for followers in the future (Deluga & Perry, 1994).

In demonstrating the effects of WFC on helping-related behavioral outcomes, this work extends an area of research that has predominantly examined how WFC affects perceptions of the target person and their careers (e.g., Carlson et al., 2008; Hoobler et al., 2009; King, 2008; Li et al., 2017a). Moreover, OCB research has typically examined intentional leadership approaches like transformational, empowering, ethical, and servant leadership (e.g., Kalshoven et al., 2016; van Dijke et al., 2012; Wang et al., 2005), which foster follower extra-role performance, often based on social exchange theory. In contrast, our results point to follower responses to what can be considered an “unintentional” leader characteristic: high leader WFC. Although leaders may feel like they need to hide their vulnerability, our work suggests they may be better off sharing with their followers when they are struggling, adding to the nascent literature on whole-self approaches at the work-family interface (e.g., Braun & Nieberle, 2017). More broadly, this work also contributes to the arguably “unintentional” effects of non-work activities and roles on work behavior and performance (e.g., Dumas & Stanko, 2017; Hammond et al., 2017).

Similarly, for the broader leadership literature, conceptualizations of leader-member exchange theory (LMX; Martin et al., 2016, 2019) and leadership styles (Chiaburu et al., 2014; Felfe & Goihl, 2002) reveal that positive leadership predicts follower motivation and willingness to go above and beyond their formal job requirements (e.g., for LMX, “I am willing to apply extra efforts beyond those normally required to help my supervisor”; Liden & Maslyn, 1998; or for servant leadership, giving back to the community; Liden et al., 2008). While LMX and positive leadership styles often reflect leaders’ intentional, work-related efforts to form relationships with and more effectively lead their followers, our results expand these views, pointing to an arguably unintentional means at the intersection of work and home through which male leaders generate more follower motivation and performance, that is, followers’ perceptions of leaders’ WFC. In doing so, we also extend research showing that family influences can energize employee effort—providing motivation or even compensating for (a lack of) motivation (Menges et al., 2017)—by showing that leaders’ families motivate follower effort in the form of interpersonal helping.

Our findings positioning male leaders, as opposed to female leaders, as the primary beneficiaries of prosocial motivation and helping in the context of WFC also offers theoretical contributions for gender role theories as well. The nascent stream of research on observers’ perceptions of others’ WFC has been explained from a gender role theory perspective: Women’s caregiving roles—which are contradictory to their commitment to the work role—confirm women’s lack-of-fit perceptions (Heilman, 2012). So, women are assumed to have higher WFC, which has harsh implications for their career evaluations and outcomes. And as we argued above, women are more likely to be type-cast as holding blame for their high WFC as compared to men high in WFC. Based on moral typecasting, men are more likely viewed as victims of work-family conflict, which prompts follower helping. In this way, we found support for a new finding of a type of caregiving premium, or perhaps better termed a ‘family conflict premium’ for male leaders. This high WFC ‘stands out’ for male leaders (Jussim et al., 1987) and prompts a like-wise extraordinary response in followers: greater prosocial motivation, dissonance, and helping.

### Practical Implications

First, our package of studies shows the conditions under which perceptions of others’ WFC trigger positive workplace effects. Participants exerted more interpersonal helping towards male leaders. This suggests a hopeful message that male leaders do not have to hide their family involvement at work and may even benefit from it, albeit pointing towards yet another potential gender bias against female leaders (see Buss et al., 2025, for a review). This complements recent research showing that family involvement—an arguably more positive aspect of family than WFC—is associated with more transformational leadership (Dumas & Stanko, 2017). This also provides a more complete picture of the effects of leaders’ work-family interface, for example: leaders and followers not only experience burnout from leaders’ actual family involvement over time (ten Brummelhuis et al., 2014), but they can also experience more cognitive dissonance, prosocial motivation, and interpersonal helping from episodic, *perceived* leader WFC.

However, long-term, it is possible that follower interpersonal helping and the resource expenditure it entails could also predict negative outcomes for followers (ten Brummelhuis et al., 2014). For example, overload or exhaustion could result from working harder, faster, or longer, even if followers’ efforts are motivated by prosocial reasons (see Bolino & Grant, 2016). Indeed, relationships are not only based on single exchanges but originate from the accumulation of multiple interactions over time. Yet, existing research on the positive outcomes of extra effort and OCB far outweighs research on its negative effects (e.g., Koopman et al., 2015).

Our results could be viewed as illuminating a bright side of WFC at work—essentially showing a positive effect from an ostensibly negative state, thereby complementing a large body of research on WFC and social support (see French et al., 2018, for a review) by highlighting followers as a specific source of instrumental support for leaders. However, when it comes to women leaders, we have uncovered a new form of gender bias at work: Male leaders receive a WFC premium that female leaders do not. In this way, leaders’ WFC is potentially yet another way in which perceptions of women in professional contexts serve to constrain their success. This may be another pathway through which patterns of professional and social status are sustained in the workplace that hold women back from achieving high performance metrics. That is, if men, who already have higher workplace status (i.e., leaders) are granted greater resources in the form of more follower help, this may hold women leaders back from the productivity necessary to ascend to the top jobs. Based on our findings, follower help likely advantages male leader performance at a higher rate than female leader performance.

By documenting gender bias in followers’ assessments of and motivation to help their leaders, we have revealed a novel mechanism of gender discrimination. Yet, knowledge of employees’ potential to hold this bias may facilitate corrective actions by organizations who wish to stem discrimination. The best ways to combat biases like this are to make employees aware (Hoobler et al., 2009) and accountable (i.e., “*the expectation that one might be asked to justify one’s action*;” Costa, 2024, p. 1673). While the initial research on organizational justice research assumed that people pursue fairness at work for relatively self-interested reasons (Cropanzano et al., 2001), later research has acknowledged a deontic justice perspective: that people are inherently interested in pursuing morality at work, treating others according to standards of right and wrong (Cropanzano et al., 2003a, 2003b). So, making followers aware of this family conflict premium for male leaders that we have uncovered may be important to accomplish during onboarding socialization or in company culture and Diversity, Equity, & Inclusion (DE&I) training sessions, or even in regular team meetings. Finally, eliminating gender bias here—ensuring women leaders can reach performance parity has direct implications for a fairer society—also helps to address gender equality (Goal #5) from the United Nations Sustainable Development Goals as well as Goal #8 which includes “*promoting sustained, inclusive, and sustainable economic growth, full and productive employment, and decent work for all*.”^9^

### Strengths, Limitations, and Future Research

We triangulated evidence of our hypothesized effects across various study methods and countries, which is rare in work-family research (see Ollier-Malaterre et al., 2013; Shockley et al., 2017). For future research, if follower help stands to benefit leaders—particularly male leaders—should leaders be encouraged to appropriately share information about their family and even family struggles at work? This could represent a specific, strategic aspect of authenticity (see Karelaia et al., 2022; Pillemer, 2024) and modern fathering (Gatrell et al., 2022) at work. Another related research question is whether helping triggered by perceived leader WFC aids leaders and organizations in the long run, but incurs negative outcomes for followers (e.g., creating or normalizing a ‘pressure to help;’ Harari et al., 2022; Koopman et al., 2020) and organizations (e.g., promoting workplace deviance via moral licensing; Loi et al., 2020). But, if having more WFC means leaders are less satisfied with their work-family balance, leaders may also respond in ways that benefit their followers directly (e.g., family-supportive leadership; Erdogan et al., 2022; Kossek et al., 2023) or indirectly (e.g., normalizing caring masculinities). These questions are ripe for future study.

Finally, it is challenging to examine cognitive dissonance in a way that does not generate endogeneity and/or substantively affect its downstream outcomes. Although we examined it in multiple ways (i.e., as ‘classic’ survey measures of guilt and discomfort as well as through LIWC- and GPT-based text analyses), because of its roots in behavior, we encourage future research to consider in-the-moment examination of cognitive dissonance in organizations via creative designs (e.g., periodic thought probes as in McVay et al., 2009). Based on our theorizing and findings, a measure tapping into guilt or a similarly more moral, emotional aspect of cognitive dissonance could be the most appropriate. But because prosocial helping is not the only path to dissonance reduction, we also encourage future studies of alternative mechanisms, such as trivialization (e.g., considering the idea that WFC is ‘not that bad’) or distraction (e.g., providing additional information that is irrelevant to the leader’s WFC; McGrath, 2017).

### Conclusion

Across three mixed-methods studies, followers responded to male leaders’ high perceived WFC in a positive, prosocial manner driven by moral cognitive dissonance, exerting more interpersonal helping towards leaders in response. This evidence reveals a behavioral family bonus wherein lower-level observers helped male (vs. female) leaders whom they ascribed more WFC, because they felt more cognitive dissonance and prosocial motivation.

## Supplementary Information

Below is the link to the electronic supplementary material.

Supplementary file1 (DOCX 2138 kb) and https://osf.io/b37xp/overview?view_only=8e28c3f69b894bf383c3bfd270455687

## Data Availability

The data that support the findings of this study are openly available on the Open Science Framework (OSF) at https://osf.io/b37xp/overview?view_only=8e28c3f69b894bf383c3bfd270455687.

## Footnotes

1 While prosocial motivation can be driven by a range of states, including egoistic and altruistic motives (e.g., empathetic concern; Batson, 1987; Batson et al., 2008), we focus on a broadly construed motivation to promote the target’s welfare (Brief & Motowidlo, 1986).

2 WFC, “*a form of inter-role conflict in which the role pressures from the work and family domains are mutually incompatible*” (Greenhaus & Beutell, 1985, p. 77). In our theorizing we use WFC to be inclusive of studies examining the opposite direction of conflict as well, that is, family-work conflict. But our research solely tests work-family conflict, often referred to as work-to-family interference.

3 A prime example is the “BBC dad,” Professor Robert Kelley, whose children crashed his television interview which went viral (i.e., 64 million views as of January 2026: https://www.youtube.com/watch?v=Mh4f9AYRCZY).

4 https://osf.io/b37xp/?view_only=ad08c046732b4aa0be84cf3293d62ed9

5 Of note, leaders’ self-reported WFC (Ω = .81) was measured with the same 3 items from Carlson et al. (2000); these scales were strongly positively correlated with each other (*r* = .66, *p* < .001). Although perceptions of leaders’ WFC has been rarely—if ever—measured by previous research, this correlation is of greater magnitude than the other sources often measured (e.g., partners and supervisors; see Junker et al., 2024).

6 Because this measurement design assessed the chronic level of followers’ general prosocial motivation toward leaders as a stable individual difference—not a specific response following an episode of perceived leader WFC—and we cannot assess temporal order in this cross-sectional survey data, we include it here as a covariate rather than as a mediator (as we do in the subsequent Studies 2 and 3) because it could be a potential suppressor in our model. Specifically, in this case, prosocial motivation explains outcome variance unrelated to our main predictors, removes noise, increases the power of the estimated effect, and makes our effect more significant while failing to absorb variance from our predictors, which is especially helpful given our smaller than expected sample; inspection of correlations confirms its status as a suppressor (see Table 1).

7 Although not hypothesized, male and female leaders’ self-reported WFC did not differ from each other, *t*(143) = 0.17, *p* = .866—a null result implicitly supporting our theorizing which also replicates previous research (e.g., King, 2008; Shockley et al., 2017).

8 Scholars have recommended using LIWC to assess cognitive dissonance (e.g., Martinie et al., 2013). LIWC allocates words to linguistic categories based on internal dictionaries. Here, we used “discrepancy”—one of the “cognition” dictionaries from the “Psychological Processes” category. This specific dictionary includes 108 words (e.g., “would,” “can,” and “could”). However, subsequent research has shown the GPT method is more accurate in detecting psychological constructs than LIWC (see Rathje et al., 2024).

9 https://www.globalgoals.org/goals/8-decent-work-and-economic-growth
