# Supplementary material for: More Motivated to Help Male Leaders? Explaining Fatherhood Bonuses via Follower Helping
Source: J Bus Ethics. 2026 Feb 11;208(2):287–307. doi: 10.1007/s10551-026-06248-1 (PMC13658328; doi:10.1007/s10551-026-06248-1)
Supplement: Supplementary file 1 [file 10551_2026_6248_MOESM1_ESM.docx]

**APPENDIX**

**STUDY 1**

**Procedure (Study 1)**

According to FlyCatcher,^[1]^ the leaders in their panel whom they contacted to participate in this study did not receive any specific instructions about which specific employee to select if they had more than one employee. They were informed about the study’s purpose and the importance of having one of their employees complete the questionnaire. Next, they requested participants provide them with the e-mail address of one employee who was at least 18 years old so that FlyCatcher could contact the employee and invite them to the questionnaire. Before proceeding, FlyCatcher asked whether the participant had asked the employee and also received permission to be contacted by FlyCatcher. If they answered “yes,” then FlyCatcher contacted the participant using the employee’s e-mail address after the leader completed their questionnaire.

**Measures (Study 1)**

**Perceived Leader Work-Family Conflict** (adapted from Carlson et al., 2000)

In general: “Strongly disagree” (1) to “Strongly agree” (5)

1. Your leader has to miss family activities due to the amount of time they must spend on work responsibilities.
2. Your leader is often so emotionally drained when they get home from work that it prevents them from contributing to their family.
3. The behaviors your leader performs that make them effective at work do not help them to be a better parent and spouse.

**Helping the Leader** (adapted from Gabriel et al., 2018; Glomb et al., 2011)

To what extent do the following statements describe how you worked with your leader after this event: “Strongly disagree” (1) to “Strongly agree” (5)

1. I volunteered to do things to help them out.
2. I worked with my leader cooperatively.
3. I spent time helping my leader with their work tasks because I wanted to.
4. I helped my leader with tasks outside the scope of work.

**OCB-Leader** (adapted from Cropanzano et al., 2003)

After this event, did you: “Strongly disagree” (1) to “Strongly agree” (7)

1. …pitch in when your leader was absent?
2. …help your leader when they had a heavy workload?
3. …assist your leader with their work when asked?
4. …take time to listen to your leader’s problems and worries?
5. …pass along information to your leader?
6. …help your leader with some of their work even if not asked?

**Results (Study 1)**

**Model Fit**

The AIC (1233.31) decreased when adding the interaction (AIC = 1223.37), suggesting improved model fit (Sakamoto et al., 1986; Vrieze, 2012); the BIC also decreased when adding the interaction (BIC = 1254.15 to 1250.16). However, the -2LL increased (from -609.66 to -602.68), meaning the interaction model did not improve raw likelihood fit. Critically, the preference shown by AIC/BIC reflects greater parsimony rather than better likelihood-based fit.

**Confirmatory Factor Analyses (CFA)**

To examine the distinctiveness of each of our measured variables, we conducted a series of CFAs using Maximum Likelihood (ML). The measurement model comprised three factors, including all participant self-reported variables with multiple items: perceived leader WFC, helping and OCB toward the leader. The three-factor model provided the best fit (χ^2^(62) = 123.03, *p* < .001; RMSEA = .083, CFI = .932, SRMR = .065), although it was not significantly better than alternative models with two factors combining the outcomes, ∆χ^2^(2) = 3.05, *p* = .218. Importantly, the 2- and 3-factors models were both a significantly better fit than a single-factor model combining all measures, ∆χ^2^(1) = 208.67, *p* < .001 and ∆χ^2^(1) = 211.72, *p* < .001, respectively. Thus, measurement discriminant validity between our predictor variable and outcome variables is empirically established. Because measurement discriminant validity between our outcome variables was not empirically established, results with the helping outcome may be the most valid based on the survey design, because participants answered about this outcome (helping the leader) before OCB toward the leader.

**Alternative Models**

```
Model A: Without Follower’s Overall Prosocial Motivation toward the Leader as a Covariate
gsem (lhelp locb <- i.lgen##c.fwfcC i.pgen), vce(robust)

Iteration 0:  Log pseudolikelihood =  -286.3854
Iteration 1:  Log pseudolikelihood =  -286.3854

Generalized structural equation model                      Number of obs = 145

Response: lhelp
Family:   Gaussian
Link:     Identity

Response: locb
Family:   Gaussian
Link:     Identity

Log pseudolikelihood = -286.3854

------------------------------------------------------------------------------
             |               Robust
             | Coefficient  std. err.      z    P>|z|     [95% conf. interval]
-------------+----------------------------------------------------------------
lhelp        |
        lgen |
       Male  |   .2535898   .1266822     2.00   0.045     .0052972    .5018824
       fwfcC |  -.1364847   .0728832    -1.87   0.061    -.2793331    .0063637
             |
lgen#c.fwfcC |
       Male  |   .2272347   .1345711     1.69   0.091    -.0365197    .4909891
             |
        pgen |
        Man  |  -.0423509   .1276583    -0.33   0.740    -.2925565    .2078547
       _cons |   3.473461   .0738684    47.02   0.000     3.328681     3.61824
-------------+----------------------------------------------------------------
locb         |
        lgen |
       Male  |   .1593746    .114972     1.39   0.166    -.0659664    .3847156
       fwfcC |  -.1324036   .0604562    -2.19   0.029    -.2508955   -.0139117
             |
lgen#c.fwfcC |
       Male  |   .1379036   .1169555     1.18   0.238    -.0913249     .367132
             |
        pgen |
        Man  |    .075565   .1089752     0.69   0.488    -.1380226    .2891525
       _cons |   3.719676   .0684925    54.31   0.000     3.585433    3.853919
-------------+----------------------------------------------------------------
 var(e.lhelp)|   .4733132   .0600664                      .3690845    .6069758
  var(e.locb)|   .3762082   .0594488                      .2760073    .5127857
------------------------------------------------------------------------------
```

```
Model B: Without any Covariates
gsem (lhelp locb <- i.lgen##c.fwfcC), vce(robust)

Iteration 0:  Log pseudolikelihood = -286.68588
Iteration 1:  Log pseudolikelihood = -286.68588

Generalized structural equation model                      Number of obs = 145

Response: lhelp
Family:   Gaussian
Link:     Identity

Response: locb
Family:   Gaussian
Link:     Identity

Log pseudolikelihood = -286.68588

------------------------------------------------------------------------------
             |               Robust
             | Coefficient  std. err.      z    P>|z|     [95% conf. interval]
-------------+----------------------------------------------------------------
lhelp        |
        lgen |
       Male  |   .2451539   .1182825     2.07   0.038     .0133246    .4769833
       fwfcC |  -.1372888   .0733434    -1.87   0.061    -.2810391    .0064616
             |
lgen#c.fwfcC |
       Male  |   .2280388   .1358556     1.68   0.093    -.0382332    .4943108
             |
       _cons |   3.460721   .0683595    50.63   0.000     3.326739    3.594703
-------------+----------------------------------------------------------------
locb         |
        lgen |
       Male  |   .1744264    .108783     1.60   0.109    -.0387844    .3876373
       fwfcC |   -.130969   .0598857    -2.19   0.029    -.2483427   -.0135952
             |
lgen#c.fwfcC |
       Male  |    .136469   .1167226     1.17   0.242     -.092303     .365241
             |
       _cons |   3.742407   .0605754    61.78   0.000     3.623681    3.861133
-------------+----------------------------------------------------------------
 var(e.lhelp)|   .4737056   .0603315                      .3690616    .6080205
  var(e.locb)|   .3774576   .0600668                      .2763198    .5156137
```

------------------------------------------------------------------------------

**STUDY 2**

**Additional Information - Methods (Study 2)**

**Helping Leaders**

As noted in our pre-registration, we planned to create two additional behavioral outcomes from participants’ feedback by counting the number of characters and the number of words in the feedback, with higher numbers indicating more effort to help the leader. However, these values were strongly, positively correlated to the point of being tantamount (i.e., *r* = .99, *p* < .001). So, we included only one of these in our measure for analysis in our summative, 3-item indicator (Ω = .88): the number of characters in the feedback (log-transformed); however, our results remain substantively unchanged if calculated with words of feedback (log-transformed) replacing characters of feedback.

Importantly, these three measures were all objectively measurable outcomes that required participants’ time and attention. However, participants did not receive extra compensation for them. Thus, ­­we view them as appropriate indicators of participants’ voluntary, (ostensibly) prosocially motivated, extra effort to help and support the leader (i.e., interpersonal helping), consistent with our theorizing.

**Additional Information - Results (Study 2)**

**Plan of Analysis**

We calculated GSEM path models in Stata (17.0). This is in contrast with the MANCOVA we mentioned in our pre-registration, because the heteroskedasticity tests we conducted were marginally significant (χ^2^[1, *N =* 420] = 3.05, *p* = .081), suggesting robust *SE*s could be useful; as our research has developed based on peer feedback and other evidence in our empirical package, prosocial motivation is also now our main theoretical mechanism rather than just a mechanism under consideration. While GSEM is a less common method to analyze data in management research, perhaps especially to analyze experimental data, GSEM and related SEM methods offer empirical advantages over more commonly used methods (e.g., ANOVA; see Bagozzi & Yi, 1989; Breitsohl, 2019), including the ability to calculate robust *SE*s which are needed here due to the threat of heteroskedasticity. GSEM and SEM also allow for simultaneous analysis of multiple equations and outcomes, but only with GSEM we can use factor notation for binary variables and interactions, which is ideal for our experimental design here with binary predictors.

**Manipulation and Comprehension Checks**

The low WFC leader (*M* = 4.43, *SD* = 1.36) was rated as having less WFC than the high WFC leader (*M* = 5.61, *SD* = 1.26), *b* = -1.19, *p* < .001, indicating that our WFC manipulation was successful.

Participants also answered three questions about the leaders in the articles: “Was the leader a man or a woman?”, “Does the leader have children?”, and “For which company does the leader work?” Participants answered an average of 2.41 (*SD* = .49) of the 3 questions correctly, indicating that they paid attention and our gender manipulation was successful.

**Examples of Study 2 Experimental Stimuli**

**Original (in German) – High WFC**

**Translation (in English, as a courtesy) – High WFC**

**Work and Life**

**No time for family time? Business leaders weigh in on modern work
and life demands**

**Families are spending little over half an hour of quality time together during the week because of the demands of modern life.**

By: A. Nezik Published: 9 January 2019

| Modern leaders are working more than ever before. Yet, German employees are often hesitant to share personal details with others at work, including private information about their feelings, family, and struggles. Leaders at large companies in particular may rarely open up about juggling jobs, children, and family.  In an interview with Vice President of BMW, Christian Fischer, who has a partner and 2 kids, said “I have to work at least 55-60 hours a week in the office, plus taking calls and answering emails, which makes it hard to maintain the relationship with my partner and kids like I would like to. Participating in all of the family activities is also not possible, which makes me feel guilty sometimes. But if I didn’t work like this now, I would have no chance for promotion in my company. Family stays most important, but I support my family through my hard work.”  He clarified, “Through hard work and engaged parenting, I show my values and lighten family integration for future generations.”  “When people say that you can have it all with so many work hours, that is just not true,” he said. “But with a supportive partner and employees, who help and care for each other, a lot is possible.” | **TOP 5 REASONS FOR**  **NOT GETTING ENOUGH**  **FAMILY TIME**  **1. Too much work**  **2. Work-related travel**  **3. Unconventional work hours**  **4. Too few good employees**  **5. An inefficient team** |
| --- | --- |

**Original (in German) – Low WFC**

**Translation (in English, as a courtesy) – Low WFC**

**Work and Life**

**Still time for the family: Business leaders weigh in on modern work
and life demands**

**Also when the challenges of modern work are high, many leaders still manage to spend several hours per day with their families.**

By: A. Nezik Published: 9 January 2019

| Modern leaders are working more than ever before. Yet, German employees are often hesitant to share personal details with others at work, including private information about their feelings, family, and struggles. Leaders at large companies in particular may rarely open up about juggling jobs, children, and family.  In an interview with Vice President of BMW, Julia Weber, who has a partner and 2 kids, said “I have to work at least 55-60 hours a week in the office, plus taking calls and answering emails, which makes it hard to maintain the relationship with my partner and kids like I would like to. Participating in all of the family activities is also not possible. But I still feel good, because I maintain a chance for promotion in my firm. Family stays most important, but I support my family through my hard work.”  She clarified, “Through hard work and engaged parenting, I show my values and lighten family integration for future generations and employees, even when the work sometimes has to wait.”  “Even when working many hours, I strongly believe that you can have a really good family life,” she said. “With a supportive partner and employees, who help and care for each other, a lot is possible.” | **TOP 5 TIPS  FOR MORE FAMILY TIME**  **1. Delegate more work**  **2. Hold meetings virtually**  **3. Work when the children are asleep**  **4. Hire productive employees**  **5. Reward efficient employees** |
| --- | --- |

**STUDY 3**

**Complete Manipulations**

**High work-family conflict.**

*“Think of a time when your leader had a high work-family conflict, or their work and family domains were incompatible.

This may include a specific event when the leader’s work kept them from family activities, when their job demands made it difficult for them to maintain the kind of relationship with their partner/children as they would like, or when they felt guilty for spending too much time or energy on work/not enough time with family.

Now take some time to write about this instance: describe your leader’s thoughts and feelings, the circumstances surrounding the event, as well as your thoughts and feelings.*

*Try to describe the event in enough detail that someone else could understand the situation by reading what you have written. (Minimum 200 characters).”*

**Control/neutral.**

*“Think of a time when you had a routine interaction with your leader that felt typical or uneventful.

This may include a specific event such as day-to-day work conversation, a regular team meeting, a task assignment, or a feedback session—anything that felt ordinary, typical, and every day.

Now take some time to write about this instance: describe your leader’s thoughts and feelings, the circumstances surrounding the event, as well as your thoughts and feelings.*

*Try to describe the event in enough detail that someone else could understand the situation by reading what you have written. (Minimum 200 characters).”*

**Measures**

Please answer the following questions in relation to yourself and how you felt about your leader in response to the event you just described.

**Discomfort** (Elliot & Devine).

The event made me feel (not at all, 1, to very much, 7):

1. Uneasy
2. Uncomfortable
3. Bothered

**Guilt** (Watson et al., 1988).

Please indicate the extent to which the event made you feel each of the following emotions (not at all, 1, to very much, 7):

1. Guilt
2. Shame

**Confirmatory Factor Analyses**

For discomfort as an indicator for dissonance, the intended 3-factor measurement model, in which all indicators loaded on their respective latent factor (i.e., discomfort, helping, OCB) had an acceptable fit (χ^2^(62) = 296.70, *p* < .001, CFI = .95, TLI = .94, RMSEA = .09, SRMR = .05). This model provided a better fit than a 2-factor model, in which helping and OCB loaded together (Δχ^2^(2) = 90.22, *p* < .001). It also had a better fit than a 1-factor model, in which all items loaded on the same factor (Δχ^2^(1) = 1706.80, *p* < .001).

For guilt as an indicator for dissonance, the corresponding 3-factor measurement model with guilt, helping, and OCB as distinct factors, also showed an acceptable fit (χ^2^(51) = 257.93, *p* < .001, CFI = .94, TLI = .93, RMSEA = .09, SRMR = .04). This model provided a better fit than a 2-factor model, in which helping and OCB loaded together (Δχ^2^(2) = 85.43, *p* < .001), and the 1-factor model (Δχ^2^(1) = 455.19, *p* < .001).

To be clear: we could not calculate these tests for the GPT and LIWC indicators of cognitive dissonance, because those are both single item measures.

**Model Fit**

The AIC (1431.37) for the main effects model decreased when adding the interaction term between leader gender and leader WFC (AIC = 1425.29), suggesting improved model fit (Sakamoto et al., 1986; Vrieze, 2012); the BIC also decreased (BIC = 1448.22 to 1446.35). Finally, the -2LL increased from -711.69 to -707.64, meaning the interaction model did not improve raw likelihood fit. Critically, the preference shown by AIC/BIC reflects greater parsimony rather than better likelihood-based fit.

**Preliminary Analyses**

In our preliminary analyses, we found evidence of heteroskedasticity (e.g., χ^2^[1, *N =* 500] = 117.03, *p* < .0001, for the interaction model of leader gender and leader work-family conflict [vs. control] predicting guilt—one of our measures of cognitive dissonance). Thus, we used robust *SE*s using the “vce(robust)” code in Stata for all models.

**Endogeneity Analyses**

Our theorized mediators (i.e., cognitive dissonance and prosocial motivation) were measured during the same experimental session as the outcome variables (i.e., helping acts and OCB toward the leader), and thus endogeneity concerns arise (Antonakis, Bendahan, Jacquart, & Lalive, 2014). Accordingly, as robustness checks, we tested whether the mediators positively predicted the different outcome measures in a series of two-stage least squares regression analyses.

To test endogeneity concerns for prosocial motivation, we used the experimental condition and marital status of leaders as instruments, as these variables significantly predicted prosocial motivation as mediator (*F* = 3.38, *p* = .018); however, these were admittedly weak instruments. The Wu-Hausman test was significant when examining the model predicting *helping* with prosocial motivation (*p* = 0.013) as well as *OCB* with prosocial motivation (*p <* .001). This indicates endogeneity concerns; furthermore, in this model, prosocial motivation did not predict helping (*B* = -0.01, *SE* = 0.34, *p* = .97) nor OCB (*B* = -0.28, *SE* = 0.37, *p* = .44). Overall, these results suggest that endogeneity bias may be present.

However, because this is our only measure of prosocial motivation and a critical piece of our moderator, and because we cannot use the experimental condition as an instrument in our complete model, we estimated an endogenous mediator model using a control‐function residual inclusion (CF–RFI) approach (Terza et al., 2008; Wooldridge, 2015). Leader care (care) and participant partner (ppartner) fulfilled the criteria for instruments for this purpose, because they were significantly correlated with prosocial motivation (*p*s = .026 - .037) but not with the other mediators or dependent variables (*p*s = .064 - .800). In the first stage, the mediator was regressed on exogenous predictors and an instrument (care/ppartner), and the resulting residuals were included in the outcome equations. However, the instrument exhibited weak predictive power in the first stage (*F* = 3.66), which limits the degree to which CF–RFI can correct for endogeneity. Accordingly, we interpret the CF–RFI estimates cautiously and present them as a robustness check alongside the primary mediation results.

To test endogeneity concerns for discomfort as an indicator of dissonance, we used the experimental condition and marital status of leaders as instruments, which jointly predicted discomfort in the first stage (*F* = 65.10, *p* < .001). The Wu-Hausman test was not significant when examining the model predicting *helping* with discomfort (*p* = 0.15), and discomfort did not significantly predict helping in the IV model (*B* = 0.10, *SE* = 0.06, *p* = .118). However, when predicting *OCB*, the Wu-Hausman test was significant (*p =* .045). But discomfort remained a significant predictor of OCB (*B* = 0.14, *SE* = 0.06, *p* = .009). Overall, these results suggest some evidence of endogeneity for discomfort; however, discomfort still functions as a robust predictor in key models.

To test endogeneity concerns for guilt as an indicator of dissonance, we used the experimental condition and marital status of leaders as instruments, which jointly predicted discomfort in the first stage (*F* = 38, *p* < .001). The Wu-Hausman test was not significant when examining the model predicting *helping* with guilt (*p* = .642), suggesting no major endogeneity issues. The effect of guilt on helping was also nonsignificant (*B* = 0.18, *p* = .087). When predicting *OCB*, the Wu-Hausmann test was again nonsignificant (*p* = .121), indicating no endogeneity problems. Guilt significantly predicted OCB in the IV model (*B* = 0.26, *SE* = 0.09, *p* = .006). Overall, the results suggest no evidence of endogeneity for guilt, supporting its use as a valid mediator.

To test endogeneity concerns for our LIWC indicator of dissonance, we used the experimental condition as an instrument, which predicted LIWC in the first stage (*F* = 12.49, *p* < .001). The Wu-Hausman test was not significant when examining the model predicting *helping* with LIWC (*p* = .137), suggesting no major endogeneity issues. The effect of LIWC on helping was significant (*B* = 1.02, *SE* = .46, *p* = .026). When predicting *OCB*, the Wu-Hausmann test was marginally significant (*p* = .059), suggesting potential endogeneity problems. LIWC significantly predicted OCB in the IV model (*B* = 1.31, *SE* = 0.51, *p* = .01). Overall, the results suggest some evidence of endogeneity for LIWC, suggesting caution for its use as a valid mediator.

Finally, to test endogeneity concerns for our GPT indicator of dissonance, we used the experimental condition as an instrument, which predicted the GPT indicator of dissonance in the first stage (*F* = 354, *p* < .001). The Wu-Hausman test was not significant when examining the model predicting *helping* with the GPT measure (*p* = .994), suggesting no major endogeneity issues. The effect of the GPT measure on helping in the IV model was significant (*B* = 0.24, *SE* = .07, *p* < .001). When predicting *OCB*, the Wu-Hausmann test was again nonsignificant (*p* = .695), indicating no endogeneity issues. The GPT measure also significantly predicted OCB in the IV model (*B* = 0.31, *SE* = 0.07, *p* < .001). These results suggest little evidence of endogeneity for the GPT measure, supporting it as a valid mediator.

**Interpretations and Conclusions**

As reported in the main text, the complete model with all four measures of cognitive dissonance showed only the GPT measure (*p* = .020) and guilt (*p* = .001) significantly predicted the second mediator: prosocial motivation; discomfort and the LIWC measure were both non-significant predictors (*p*s = .462 - .871). Furthermore, neither discomfort nor LIWC consistently and significantly predicted our interpersonal helping outcomes (*p*s = .022 - .155 for LIWC; *p*s = .624 - .993 for discomfort). Thus, we conduct our moderated serial mediation model calculated only with the GPT and guilt measures.

Taken together, these empirical results suggest that the cognitive dissonance (GPT) measure is a stronger mediator because it shows a lack of endogeneity (similar to what we found for guilt) but it predicts both helping and OCB (guilt only predicts OCB). However, the GPT measure revealed negative associations with prosocial motivation and indirect effects, while the guilt measure revealed positive associations with prosocial motivation and indirect effects (as we theorized and pre-registered).

After closer inspection and reflection, although these two measures of dissonance correlate highly (*r* = .78), they are conceptually not interchangeable. Instead, our GPT measure captures a cold, cognitive mismatch or conflict state, whereas our guilt measure captures a hot, moralized, interpersonal concern. That is, our GPT measure is also capturing followers’ perceived responsibility, recognition of the leader’s struggles, and awareness of conflict in the leader’s situation, which can reflect a direct effect which is not motivational but rather a form of behavioral compliance based on normative or obligation-driven helping. In contrast, guilt is a classic prosocial emotion that increases concern for others and motivates repair, enhancing followers’ desire to act in ways that could help the leader. For these reasons, while both may be empirically valid mediators in our model and related but slightly different indicator of cognitive dissonance—and even the GPT measure is empirically a bit stronger here—based on our pre-registration and moral theorizing, we emphasize the findings for guilt.

**Alternate Models**

**MODEL A**: Combined model with all four cognitive dissonance variables

| sem /// | |  | | | | | | |  |
| --- | --- | --- | --- | --- | --- | --- | --- | --- | --- |
| > (cogdisC <- wfcD genL wfcXgenL) /// | | | | | |  |  |  |  |
| > (guiltC <- wfcD genL wfcXgenL) /// | | | | | |  |  |  |  |
| > (discomfortC <- wfcD genL wfcXgenL) /// | | | | | | |  |  |  |
| > (liwcC <- wfcD genL wfcXgenL) /// | | | | | |  |  |  |  |
| > (prosoC <- cogdisC guiltC discomfortC liwcC wfcD genL wfcXgenL) /// | | | | | | | | | |
| > (helping <- prosoC cogdisC guiltC discomfortC liwcC wfcD genL wfcXgenL) /// | | | | | | | | | |
| > (ocb <- prosoC cogdisC guiltC discomfortC liwcC wfcD genL wfcXgenL), /// | | | | | | | | | |
| > vce(robust) | | |  |  |  |  |  |  |  |
|  |  |  |  |  |  |  |  |  |  |
|  |  |  |  |  |  |  |  |  |  |
| Endogenous variables | | |  |  |  |  |  |  |  |
| Observed: cogdisC guiltC discomfortC liwcC prosoC helping ocb | | | | | | | |  |  |
|  |  |  |  |  |  |  |  |  |  |
| Exogenous variables | | |  |  |  |  |  |  |  |
| Observed: wfcD genL wfcXgenL | | | |  |  |  |  |  |  |
|  |  |  |  |  |  |  |  |  |  |
| Fitting target model: | | |  |  |  |  |  |  |  |
| Iteration 0: Log pseudolikelihood = -5362.7484 | | | | | | |  |  |  |
| Iteration 1: Log pseudolikelihood = -5362.7484 | | | | | | |  |  |  |
|  |  |  |  |  |  |  |  |  |  |
| Structural equation model Number of obs = 499 | | | | | | | | | |
| Estimation method: ml | | |  |  |  |  |  |  |  |
|  |  |  |  |  |  |  |  |  |  |
| Log pseudolikelihood = -5362.7484 | | | | |  |  |  |  |  |
|  |  |  |  |  |  |  |  |  |  |
| ----------------------------------------------------------------------------------- | | | | | | | | | |
| \| Robust | | | | | |  |  |  |  |
| \| Coefficient std. err. z P>\|z\| [95% conf. interval] | | | | | | | | | |
| ------------------+---------------------------------------------------------------- | | | | | | | | | |
| Structural \| | | |  |  |  |  |  |  |  |
| cogdisC \| | | |  |  |  |  |  |  |  |
| wfcD \| 1.079398 .0946272 11.41 0.000 .8939324 1.264864 | | | | | | | | | |
| genL \| .1126275 .0824642 1.37 0.172 -.0489993 .2742544 | | | | | | | | | |
| wfcXgenL \| .3830146 .1346423 2.84 0.004 .1191206 .6469086 | | | | | | | | | |
| _cons \| -.6758459 .0607926 -11.12 0.000 -.7949973 -.5566945 | | | | | | | | | |
| ----------------+---------------------------------------------------------------- | | | | | | | | | |
| guiltC \| | | |  |  |  |  |  |  |  |
| wfcD \| .6658983 .0947814 7.03 0.000 .4801301 .8516665 | | | | | | | | | |
| genL \| .1563845 .0712669 2.19 0.028 .0167039 .2960652 | | | | | | | | | |
| wfcXgenL \| .3845054 .1581911 2.43 0.015 .0744566 .6945542 | | | | | | | | | |
| _cons \| -.5009604 .0457922 -10.94 0.000 -.5907114 -.4112094 | | | | | | | | | |
| ----------------+---------------------------------------------------------------- | | | | | | | | | |
| discomfortC \| | | |  |  |  |  |  |  |  |
| wfcD \| .8484077 .1109687 7.65 0.000 .630913 1.065902 | | | | | | | | | |
| genL \| .0718326 .09155 0.78 0.433 -.1076022 .2512673 | | | | | | | | | |
| wfcXgenL \| .4086432 .1509591 2.71 0.007 .1127688 .7045177 | | | | | | | | | |
| _cons \| -.5531618 .0699709 -7.91 0.000 -.6903023 -.4160213 | | | | | | | | | |
| ----------------+---------------------------------------------------------------- | | | | | | | | | |
| liwcC \| | | |  |  |  |  |  |  |  |
| wfcD \| .3241462 .1435701 2.26 0.024 .042754 .6055384 | | | | | | | | | |
| genL \| -.204399 .1156486 -1.77 0.077 -.4310662 .0222681 | | | | | | | | | |
| wfcXgenL \| -.0383924 .1802757 -0.21 0.831 -.3917263 .3149415 | | | | | | | | | |
| _cons \| -.0342053 .0904978 -0.38 0.705 -.2115777 .1431671 | | | | | | | | | |
| ----------------+---------------------------------------------------------------- | | | | | | | | | |
| prosoC \| | | |  |  |  |  |  |  |  |
| cogdisC \| -.4080362 .175503 -2.32 0.020 -.7520158 -.0640567 | | | | | | | | | |
| guiltC \| .3177434 .0934656 3.40 0.001 .1345542 .5009325 | | | | | | | | | |
| discomfortC \| -.0209886 .1293727 -0.16 0.871 -.2745545 .2325772 | | | | | | | | | |
| liwcC \| -.0355672 .0483205 -0.74 0.462 -.1302737 .0591393 | | | | | | | | | |
| wfcD \| .1249058 .1696609 0.74 0.462 -.2076234 .457435 | | | | | | | | | |
| genL \| -.0407291 .1159381 -0.35 0.725 -.2679636 .1865054 | | | | | | | | | |
| wfcXgenL \| .0665312 .1742238 0.38 0.703 -.2749411 .4080035 | | | | | | | | | |
| _cons \| -.0543481 .1105179 -0.49 0.623 -.2709592 .162263 | | | | | | | | | |
| ----------------+---------------------------------------------------------------- | | | | | | | | | |
| helping \| | | |  |  |  |  |  |  |  |
| cogdisC \| .1783214 .1751988 1.02 0.309 -.165062 .5217048 | | | | | | | | | |
| guiltC \| .0236305 .0808731 0.29 0.770 -.1348779 .1821389 | | | | | | | | | |
| discomfortC \| -.0011583 .1237435 -0.01 0.993 -.2436912 .2413745 | | | | | | | | | |
| liwcC \| -.0843534 .0592903 -1.42 0.155 -.2005603 .0318534 | | | | | | | | | |
| prosoC \| .8894089 .0565923 15.72 0.000 .7784901 1.000328 | | | | | | | | | |
| wfcD \| .0373339 .1590039 0.23 0.814 -.2743079 .3489758 | | | | | | | | | |
| genL \| -.1344235 .1322148 -1.02 0.309 -.3935597 .1247128 | | | | | | | | | |
| wfcXgenL \| .0807215 .1864013 0.43 0.665 -.2846184 .4460614 | | | | | | | | | |
| _cons \| 5.145394 .0993597 51.79 0.000 4.950652 5.340135 | | | | | | | | | |
| ----------------+---------------------------------------------------------------- | | | | | | | | | |
| ocb \| | | |  |  |  |  |  |  |  |
| cogdisC \| .1799368 .1592182 1.13 0.258 -.1321252 .4919988 | | | | | | | | | |
| guiltC \| -.0413961 .0687388 -0.60 0.547 -.1761216 .0933294 | | | | | | | | | |
| discomfortC \| .0551761 .1126562 0.49 0.624 -.1656259 .2759782 | | | | | | | | | |
| liwcC \| -.1101351 .0480635 -2.29 0.022 -.2043378 -.0159325 | | | | | | | | | |
| prosoC \| .8055488 .0529374 15.22 0.000 .7017934 .9093042 | | | | | | | | | |
| wfcD \| .0918735 .161081 0.57 0.568 -.2238395 .4075865 | | | | | | | | | |
| genL \| -.1236274 .126743 -0.98 0.329 -.372039 .1247843 | | | | | | | | | |
| wfcXgenL \| .1496211 .1693116 0.88 0.377 -.1822235 .4814658 | | | | | | | | | |
| _cons \| 5.311304 .1068174 49.72 0.000 5.101946 5.520663 | | | | | | | | | |
| ------------------+---------------------------------------------------------------- | | | | | | | | | |
| var(e.cogdisC)\| .5560096 .0377389 .4867516 .6351221 | | | | | | | | | |
| var(e.guiltC)\| .7735362 .0653361 .655518 .9128023 | | | | | | | | | |
| var(e.discomfortC)\| .6885471 .0440536 .607398 .7805379 | | | | | | | | | |
| var(e.liwcC)\| .9665804 .0888119 .8072859 1.157307 | | | | | | | | | |
| var(e.prosoC)\| .93808 .0730762 .8052515 1.092819 | | | | | | | | | |
| var(e.helping)\| 1.110005 .0867639 .9523364 1.293777 | | | | | | | | | |
| var(e.ocb)\| .8964554 .083802 .7463751 1.076714 | | | | | | | | | |
| ----------------------------------------------------------------------------------- | | | | | | | | | |

**MODEL B: Only the two significant cognitive dissonance variables but including covariates (i.e., number of words written, perceived acceptability of overtime, and participant gender)**

| . sem /// | |  | | | | | | | |
| --- | --- | --- | --- | --- | --- | --- | --- | --- | --- |
| > (cogdisC <- wfcD genL wfcXgenL overtime words pgen) /// | | | | | | | |  |  |
| > (guiltC <- wfcD genL wfcXgenL overtime words pgen) /// | | | | | | | |  |  |
| > (prosoC <- cogdisC guiltC wfcD genL wfcXgenL overtime words pgen) /// | | | | | | | | |  |
| > (helping <- prosoC cogdisC guiltC wfcD genL wfcXgenL overtime words pgen) /// | | | | | | | | | |
| > (ocb <- prosoC cogdisC guiltC wfcD genL wfcXgenL overtime words pgen), /// | | | | | | | | | |
| > vce(robust) | | |  |  |  |  |  |  |  |
|  |  |  |  |  |  |  |  |  |  |
|  |  |  |  |  |  |  |  |  |  |
| Endogenous variables | | |  |  |  |  |  |  |  |
| Observed: cogdisC guiltC prosoC helping ocb | | | | | |  |  |  |  |
|  |  |  |  |  |  |  |  |  |  |
| Exogenous variables | | |  |  |  |  |  |  |  |
| Observed: wfcD genL wfcXgenL overtime words pgen | | | | | |  |  |  |  |
|  |  |  |  |  |  |  |  |  |  |
| Fitting target model: | | |  |  |  |  |  |  |  |
| Iteration 0: Log pseudolikelihood = -8167.9083 | | | | | |  |  |  |  |
| Iteration 1: Log pseudolikelihood = -8167.9083 | | | | | |  |  |  |  |
|  |  |  |  |  |  |  |  |  |  |
| Structural equation model Number of obs = 497 | | | | | | | | |  |
| Estimation method: ml | | |  |  |  |  |  |  |  |
|  |  |  |  |  |  |  |  |  |  |
| Log pseudolikelihood = -8167.9083 | | | |  |  |  |  |  |  |
|  |  |  |  |  |  |  |  |  |  |
| ------------------------------------------------------------------------------- | | | | | | | | |  |
| \| Robust | | | | |  |  |  |  |  |
| \| Coefficient std. err. z P>\|z\| [95% conf. interval] | | | | | | | | |  |
| --------------+---------------------------------------------------------------- | | | | | | | | |  |
| Structural \| | |  |  |  |  |  |  |  |  |
| cogdisC \| | |  |  |  |  |  |  |  |  |
| wfcD \| .844665 .1309881 6.45 0.000 .5879331 1.101397 | | | | | | | | |  |
| genL \| .096738 .0880336 1.10 0.272 -.0758048 .2692807 | | | | | | | | |  |
| wfcXgenL \| .3507312 .1338542 2.62 0.009 .0883818 .6130806 | | | | | | | | |  |
| overtime \| -.0651001 .0353461 -1.84 0.066 -.1343772 .0041769 | | | | | | | | |  |
| words \| .0007236 .0002485 2.91 0.004 .0002366 .0012106 | | | | | | | | |  |
| pgen \| .0479356 .0719314 0.67 0.505 -.0930473 .1889185 | | | | | | | | |  |
| _cons \| -.5173924 .1389058 -3.72 0.000 -.7896427 -.245142 | | | | | | | | |  |
| ------------+---------------------------------------------------------------- | | | | | | | | |  |
| guiltC \| | |  |  |  |  |  |  |  |  |
| wfcD \| .5838448 .169336 3.45 0.001 .2519524 .9157372 | | | | | | | | |  |
| genL \| .0880479 .0797721 1.10 0.270 -.0683026 .2443984 | | | | | | | | |  |
| wfcXgenL \| .3738611 .1581958 2.36 0.018 .063803 .6839193 | | | | | | | | |  |
| overtime \| -.0294099 .0431629 -0.68 0.496 -.1140077 .0551879 | | | | | | | | |  |
| words \| .0002607 .0003961 0.66 0.511 -.0005157 .0010371 | | | | | | | | |  |
| pgen \| .1488886 .0803011 1.85 0.064 -.0084986 .3062757 | | | | | | | | |  |
| _cons \| -.4592962 .1645076 -2.79 0.005 -.7817251 -.1368673 | | | | | | | | |  |
| ------------+---------------------------------------------------------------- | | | | | | | | |  |
| prosoC \| | |  |  |  |  |  |  |  |  |
| cogdisC \| -.4646075 .1108149 -4.19 0.000 -.6818008 -.2474142 | | | | | | | | |  |
| guiltC \| .3370705 .0886656 3.80 0.000 .1632892 .5108518 | | | | | | | | |  |
| wfcD \| -.1395939 .1779418 -0.78 0.433 -.4883534 .2091656 | | | | | | | | |  |
| genL \| -.0441305 .1232978 -0.36 0.720 -.2857898 .1975287 | | | | | | | | |  |
| wfcXgenL \| .035428 .1717963 0.21 0.837 -.3012866 .3721426 | | | | | | | | |  |
| overtime \| .1617104 .0517422 3.13 0.002 .0602976 .2631233 | | | | | | | | |  |
| words \| .0008937 .0002688 3.32 0.001 .0003668 .0014206 | | | | | | | | |  |
| pgen \| -.0157312 .0984483 -0.16 0.873 -.2086863 .177224 | | | | | | | | |  |
| _cons \| -.7112695 .2257021 -3.15 0.002 -1.153638 -.2689014 | | | | | | | | |  |
| ------------+---------------------------------------------------------------- | | | | | | | | |  |
| helping \| | |  |  |  |  |  |  |  |  |
| cogdisC \| .1515737 .0890569 1.70 0.089 -.0229746 .3261221 | | | | | | | | |  |
| guiltC \| .0331477 .0728323 0.46 0.649 -.1096011 .1758964 | | | | | | | | |  |
| prosoC \| .8626559 .0589791 14.63 0.000 .747059 .9782527 | | | | | | | | |  |
| wfcD \| -.0776598 .1926597 -0.40 0.687 -.455266 .2999463 | | | | | | | | |  |
| genL \| -.1537378 .1427641 -1.08 0.282 -.4335502 .1260746 | | | | | | | | |  |
| wfcXgenL \| .0796589 .1858452 0.43 0.668 -.284591 .4439087 | | | | | | | | |  |
| overtime \| .1076896 .0583554 1.85 0.065 -.0066849 .2220641 | | | | | | | | |  |
| words \| .000333 .0003317 1.00 0.315 -.0003171 .0009831 | | | | | | | | |  |
| pgen \| .0307737 .1107555 0.28 0.781 -.1863031 .2478504 | | | | | | | | |  |
| _cons \| 4.729696 .2306906 20.50 0.000 4.27755 5.181841 | | | | | | | | |  |
| ------------+---------------------------------------------------------------- | | | | | | | | |  |
| ocb \| | |  |  |  |  |  |  |  |  |
| cogdisC \| .2143043 .0809983 2.65 0.008 .0555506 .373058 | | | | | | | | |  |
| guiltC \| -.0422769 .062145 -0.68 0.496 -.1640788 .079525 | | | | | | | | |  |
| prosoC \| .7703939 .0546322 14.10 0.000 .6633167 .8774711 | | | | | | | | |  |
| wfcD \| -.0873384 .1780378 -0.49 0.624 -.4362861 .2616094 | | | | | | | | |  |
| genL \| -.1149008 .1386799 -0.83 0.407 -.3867084 .1569067 | | | | | | | | |  |
| wfcXgenL \| .1517891 .1671764 0.91 0.364 -.1758707 .4794488 | | | | | | | | |  |
| overtime \| .1500382 .0536388 2.80 0.005 .044908 .2551683 | | | | | | | | |  |
| words \| .0004557 .0002652 1.72 0.086 -.000064 .0009754 | | | | | | | | |  |
| pgen \| -.0353782 .1025142 -0.35 0.730 -.2363024 .165546 | | | | | | | | |  |
| _cons \| 4.762829 .2228669 21.37 0.000 4.326018 5.19964 | | | | | | | | |  |
| --------------+---------------------------------------------------------------- | | | | | | | | |  |
| var(e.cogdisC)\| .5418421 .037596 .4729462 .6207745 | | | | | | | | |  |
| var(e.guiltC)\| .7687999 .0652347 .6510084 .9079043 | | | | | | | | |  |
| var(e.prosoC)\| .8928104 .0703211 .7650952 1.041845 | | | | | | | | |  |
| var(e.helping)\| 1.101459 .0859832 .945194 1.283559 | | | | | | | | |  |
| var(e.ocb)\| .8815859 .0818653 .7348874 1.057568 | | | | | | | | |  |
| ------------------------------------------------------------------------------- | | | | | | | | |  |

**MODEL C: Only the two significant cognitive dissonance variables with the endogeneity control (**v_prosoCint2sig)

| . sem /// | |  | | | | |  |  |  |
| --- | --- | --- | --- | --- | --- | --- | --- | --- | --- |
| > (cogdisC <- wfcD genL wfcXgenL) /// | | | | | |  |  |  |  |
| > (guiltC <- wfcD genL wfcXgenL) /// | | | | |  |  |  |  |  |
| > (prosoC <- cogdisC guiltC wfcD genL wfcXgenL) /// | | | | | | |  |  |  |
| > (helping <- prosoC cogdisC guiltC wfcD genL wfcXgenL v_prosoCint2sig) /// | | | | | | | | | |
| > (ocb <- prosoC cogdisC guiltC wfcD genL wfcXgenL v_prosoCint2sig), /// | | | | | | | | |  |
| > vce(robust) | | |  |  |  |  |  |  |  |
|  |  |  |  |  |  |  |  |  |  |
|  |  |  |  |  |  |  |  |  |  |
| Endogenous variables | | |  |  |  |  |  |  |  |
| Observed: cogdisC guiltC prosoC helping ocb | | | | | |  |  |  |  |
|  |  |  |  |  |  |  |  |  |  |
| Exogenous variables | | |  |  |  |  |  |  |  |
| Observed: wfcD genL wfcXgenL v_prosoCint2sig | | | | | |  |  |  |  |
|  |  |  |  |  |  |  |  |  |  |
| Fitting target model: | | |  |  |  |  |  |  |  |
| Iteration 0: Log pseudolikelihood = -4741.8005 | | | | | |  |  |  |  |
| Iteration 1: Log pseudolikelihood = -4741.8005 | | | | | |  |  |  |  |
|  |  |  |  |  |  |  |  |  |  |
| Structural equation model Number of obs = 499 | | | | | | | | |  |
| Estimation method: ml | | |  |  |  |  |  |  |  |
|  |  |  |  |  |  |  |  |  |  |
| Log pseudolikelihood = -4741.8005 | | | |  |  |  |  |  |  |
|  |  |  |  |  |  |  |  |  |  |
| ----------------------------------------------------------------------------------- | | | | | | | | | |
| \| Robust | | | | |  |  |  |  |  |
| \| Coefficient std. err. z P>\|z\| [95% conf. interval] | | | | | | | | | |
| ------------------+---------------------------------------------------------------- | | | | | | | | | |
| Structural \| | | |  |  |  |  |  |  |  |
| cogdisC \| | | |  |  |  |  |  |  |  |
| wfcD \| 1.079398 .0946272 11.41 0.000 .8939324 1.264864 | | | | | | | | | |
| genL \| .1126275 .0824642 1.37 0.172 -.0489993 .2742544 | | | | | | | | | |
| wfcXgenL \| .3830146 .1346423 2.84 0.004 .1191206 .6469086 | | | | | | | | | |
| _cons \| -.6758459 .0607926 -11.12 0.000 -.7949973 -.5566945 | | | | | | | | | |
| ----------------+---------------------------------------------------------------- | | | | | | | | | |
| guiltC \| | | |  |  |  |  |  |  |  |
| wfcD \| .6658983 .0947814 7.03 0.000 .4801301 .8516665 | | | | | | | | | |
| genL \| .1563845 .0712669 2.19 0.028 .0167039 .2960652 | | | | | | | | | |
| wfcXgenL \| .3845054 .1581911 2.43 0.015 .0744566 .6945542 | | | | | | | | | |
| _cons \| -.5009604 .0457922 -10.94 0.000 -.5907114 -.4112094 | | | | | | | | | |
| ----------------+---------------------------------------------------------------- | | | | | | | | | |
| prosoC \| | | |  |  |  |  |  |  |  |
| cogdisC \| -.4379492 .1104381 -3.97 0.000 -.6544039 -.2214945 | | | | | | | | | |
| guiltC \| .3255502 .0913499 3.56 0.000 .1465077 .5045928 | | | | | | | | | |
| wfcD \| .1226594 .1675751 0.73 0.464 -.2057817 .4511005 | | | | | | | | | |
| genL \| -.0328187 .1171167 -0.28 0.779 -.2623633 .1967258 | | | | | | | | | |
| wfcXgenL \| .0677752 .174793 0.39 0.698 -.2748127 .4103631 | | | | | | | | | |
| _cons \| -.057827 .1122711 -0.52 0.607 -.2778742 .1622202 | | | | | | | | | |
| ----------------+---------------------------------------------------------------- | | | | | | | | | |
| helping \| | | |  |  |  |  |  |  |  |
| cogdisC \| .2710314 .2023567 1.34 0.180 -.1255805 .6676432 | | | | | | | | | |
| guiltC \| -.0504837 .1493256 -0.34 0.735 -.3431566 .2421892 | | | | | | | | | |
| prosoC \| 1.126071 .4030316 2.79 0.005 .336144 1.915999 | | | | | | | | | |
| wfcD \| -.010168 .1710461 -0.06 0.953 -.3454123 .3250763 | | | | | | | | | |
| genL \| -.1087247 .1321107 -0.82 0.411 -.3676569 .1502075 | | | | | | | | | |
| wfcXgenL \| .0705084 .1877042 0.38 0.707 -.297385 .4384019 | | | | | | | | | |
| v_prosoCint2sig \| -.2372187 .4028802 -0.59 0.556 -1.026849 .552412 | | | | | | | | | |
| _cons \| 5.156683 .1037933 49.68 0.000 4.953252 5.360114 | | | | | | | | | |
| ----------------+---------------------------------------------------------------- | | | | | | | | | |
| ocb \| | | |  |  |  |  |  |  |  |
| cogdisC \| .3295803 .1791886 1.84 0.066 -.0216229 .6807835 | | | | | | | | | |
| guiltC \| -.1255814 .1279719 -0.98 0.326 -.3764016 .1252388 | | | | | | | | | |
| prosoC \| 1.020085 .3502788 2.91 0.004 .3335514 1.706619 | | | | | | | | | |
| wfcD \| .0261126 .1724134 0.15 0.880 -.3118114 .3640366 | | | | | | | | | |
| genL \| -.0941405 .1274865 -0.74 0.460 -.3440095 .1557285 | | | | | | | | | |
| wfcXgenL \| .1460424 .1678984 0.87 0.384 -.1830324 .4751172 | | | | | | | | | |
| v_prosoCint2sig \| -.2139292 .3504745 -0.61 0.542 -.9008466 .4729882 | | | | | | | | | |
| _cons \| 5.327407 .1112054 47.91 0.000 5.109448 5.545366 | | | | | | | | | |
| ------------------+---------------------------------------------------------------- | | | | | | | | | |
| var(e.cogdisC)\| .5560096 .0377389 .4867516 .6351221 | | | | | | | | | |
| var(e.guiltC)\| .7735362 .0653361 .655518 .9128023 | | | | | | | | | |
| var(e.prosoC)\| .9393166 .0732382 .8062025 1.09441 | | | | | | | | | |
| var(e.helping)\| 1.11605 .0874286 .9572002 1.301262 | | | | | | | | | |
| var(e.ocb)\| .9081973 .0840703 .7575058 1.088866 | | | | | | | | | |
| ----------------------------------------------------------------------------------- | | | | | | | | | |

**APPENDIX REFERENCES**

Antonakis, J., Bendahan, S., Jacquart, P., & Lalive, R. 2014. Causality and Endogeneity: Problems and Solutions. *The Oxford Handbook of Leadership and Organizations*.

Bagozzi, R. P., & Yi, Y. (1989). On the use of structural equation models in experimental

designs. *Journal of Marketing Research, 26*(3), 271-284.

Boyd, R. L., Ashokkumar, A., Seraj, S., & Pennebaker, J. W. (2022). *The development*

*and psychometric properties of LIWC-22*. Austin, TX: University of Texas at Austin.

<https://www.liwc.app>

Brand, J., Israeli, A., & Ngwe, D. (2023). Using LLMs for market research. *HBS Working Paper*, (23-062).

Breitsohl, H. (2019). Beyond ANOVA: An introduction to structural equation models for experimental designs. *Organizational Research Methods, 22*(3), 649-677.

Carlson, D. S., Kacmar, K. M., & Williams, L. J. (2000). Construction and initial validation of a multidimensional measure of WFC. *Journal of Vocational Behavior*, *56*, 249–276.

Cropanzano, R., Rupp, D. E., & Byrne, Z. S. (2003). The relationship of emotional
exhaustion to work attitudes, performance, and organizational citizenship behaviors.	*Journal of Applied Psychology*, 88(1), 160-169.

Festinger, L. (1962). Cognitive Dissonance. *Scientific American*, *207*(4), 93–106.

Gabriel, A. S., Koopman, J., Rosen, C. C., & Johnson, R. E. (2018). Helping others or
helping oneself? An episodic examination of the behavioural consequences of helping	at work. *Personnel Psychology*, 71, 85-107.

Glauber, R. (2008). Race and gender in families and at work: The fatherhood wage

premium. *Gender & Society*, *22*(1), 8-30.

Glomb, T. M., Bhave, D. P., Miner, A. G., & Wall, M. (2011). Doing good, feeling good:

Examining the role of organizational citizenship behaviors in changing mood. *Personnel Psychology*, *64*(1), 191-223.

Greenhaus, J. H., & Beutell, N. J. (1985). Sources of conflict between work and family roles. *Academy of Management Review*, *10*(1), 76–88.

Grimes, M., Von Krogh, G., Feuerriegel, S., Rink, F., & Gruber, M. (2023). From scarcity to abundance: Scholars and scholarship in an age of generative artificial intelligence. *Academy of Management Journal*, *66*(6), 1617-1624.

Grube, A., Schroer, J., Hentzschel, C., & Hertel, G. (2008). The event reconstruction method: An efficient measure of experienced-based job satisfaction. *Journal of Occupational and Organizational Psychology*, *81*, 669–689.

Hewitt, L., Ashokkumar, A., Ghezae, I., & Willer, R. (2024). Predicting results of social science experiments using large language models. *Proceedings of the National Academy of Sciences.*

Hodges, M. J., & Budig, M. J. (2010). Who gets the daddy bonus? Organizational hegemonic

masculinity and the impact of fatherhood on earnings. *Gender & Society*, *24*(6), 717-

745.

Kahneman, D., Krueger, A. B., Schkade, D. A., Schwarz, N., & Stone, A. A. (2004). A survey method for characterizing daily life experience. *Science*, *5702*, 1776–1780.

Li, P., Castelo, N., Katona, Z., & Sarvary, M. (2024). Frontiers: Determining the validity of large language models for automated perceptual analysis. Marketing Science, *43*(2), 239–468.

Manning, B. S., Zhu, K., & Horton, J. J. (2024). Automated social science: Language models as scientist and subjects. National Bureau of Economic Research (Working Paper 32381).

Martinie, M., Milland, L., & Olive, T. (2013). Some theoretical considerations on attitude, arousal, and affect during cognitive dissonance. *Social and Personality Psychology Compass*, *7*(9), 680–688.

OpenAI. (2024). ChatGPT [Large language model]. Retrieved November 2024, from <https://chatgpt.com>

Padavic, I., Ely, R. J., & Reid, E. M. (2020). Explaining the persistence of gender inequality, The work-family narrative as a social defense against the 24/7 work culture. *Administrative Science Quarterly*, *65*(1), 61–111.

Podsakoff, P. M., & Podsakoff, N. P. (2019). Experimental designs in management and

leadership research: Strengths, limitations, and recommendations for improving publishability. *The Leadership Quarterly, 30*(1), 11-33.

Rathje, S., Mirea, D.-M., Sucholutsky, I., Marjieh, R., Robertson, C. E., & Van Bavel, J. J.

(2024). GPT is an effective tool for multilingual psychological text analysis.

*Proceedings of the National Academy of Sciences*, *121*(34), e2308950121.

Reid, E. (2015). Embracing, passing, revealing, and the ideal worker image: How people

navigate expected and experienced professional identities. *Organization*

*Science*, *26*(4), 997-1017.

Rubenstein, A. L., Kammeyer-Mueller, J. D., & Thundiyil, T. G. (2020). The comparative

effects of supervisor helping motives on newcomer adjustment and socialization

outcomes. *Journal of Applied Psychology*, 105(12), 1466-1489.

Sakamoto, Y., Ishiguro, M., and Kitagawa G. (1986). Akaike Information Criterion Statistics.

D. Reidel Publishing Company.

Terza, J. V., Basu, A., & Rathouz, P. J. (2008). Two-stage residual inclusion estimation:

Addressing endogeneity in health econometric modeling. *Journal of Health*

*Economics, 27*(3), 531–543.

Vrieze, S. I. (2012). Model selection and psychological theory: A discussion of the

differences between the Akaike Information Criterion (AIC) and the Bayesian

Information Criterion (BIC). *Psychological Methods*, 17(2), 228-243.

Wooldridge, J. M. (2015). Control function methods in applied econometrics. *The* *Journal of*

*Human Resources, 50*(2), 420–445.

1. For more details, see here: [Research - Flycatcher internet research](https://www.flycatcher.eu/en/Home/Research)
